# β-Hairpin-Mediated Formation of Structurally Distinct Multimers of Neurotoxic Prion Peptides

**DOI:** 10.1371/journal.pone.0087354

**Published:** 2014-01-31

**Authors:** Andrew C. Gill

**Affiliations:** The Roslin Institute and Royal (Dick) School of Veterinary Studies, Easter Bush Campus, University of Edinburgh, Roslin, Edinburgh, United Kingdom; Rocky Mountain Laboratories, NIAID, NIH, United States of America

## Abstract

Protein misfolding disorders are associated with conformational changes in specific proteins, leading to the formation of potentially neurotoxic amyloid fibrils. During pathogenesis of prion disease, the prion protein misfolds into β-sheet rich, protease-resistant isoforms. A key, hydrophobic domain within the prion protein, comprising residues 109–122, recapitulates many properties of the full protein, such as helix-to-sheet structural transition, formation of fibrils and cytotoxicity of the misfolded isoform. Using all-atom, molecular simulations, it is demonstrated that the monomeric 109–122 peptide has a preference for α-helical conformations, but that this peptide can also form β-hairpin structures resulting from turns around specific glycine residues of the peptide. Altering a single amino acid within the 109–122 peptide (A_117_V, associated with familial prion disease) increases the prevalence of β-hairpin formation and these observations are replicated in a longer peptide, comprising residues 106–126. Multi-molecule simulations of aggregation yield different assemblies of peptide molecules composed of conformationally-distinct monomer units. Small molecular assemblies, consistent with oligomers, comprise peptide monomers in a β-hairpin-like conformation and in many simulations appear to exist only transiently. Conversely, larger assemblies are comprised of extended peptides in predominately antiparallel β-sheets and are stable relative to the length of the simulations. These larger assemblies are consistent with amyloid fibrils, show cross-β structure and can form through elongation of monomer units within pre-existing oligomers. In some simulations, assemblies containing both β-hairpin and linear peptides are evident. Thus, in this work oligomers are on pathway to fibril formation and a preference for β-hairpin structure should enhance oligomer formation whilst inhibiting maturation into fibrils. These simulations provide an important new atomic-level model for the formation of oligomers and fibrils of the prion protein and suggest that stabilization of β-hairpin structure may enhance cellular toxicity by altering the balance between oligomeric and fibrillar protein assemblies.

## Introduction

The assembly of specific protein molecules into amyloid fibrils underlies several devastating neurodegenerative disorders, including Alzheimer’s disease, Parkinson’s disease, Huntington’s disease, amyotrophic lateral sclerosis and prion diseases [1]. The formation of amyloid by protein misfolding is also believed to be causative in a variety of non-neurodegenerative clinical syndromes, such as type II diabetes [2]. The proteins within amyloid fibrils are in predominately β-strand conformation and are organized into large β-sheets; the β-strands run perpendicular to the axis of the fibril, giving rise to the “cross-β” architecture that is characteristic of amyloid. Many different proteins may contain short sections with high propensity to form amyloid [3], however, recent investigations suggest that small, oligomeric or protofibrillar species, which are structurally different to fibrils [4], [5] are actually responsible for the bulk of the cellular toxicity observed in protein misfolding diseases [6], [7], [8], [9]. It is critically important that we determine the molecular mechanisms by which specific, normal, cellular proteins, which comprise proteins from a disparate group of sequences and cellular locations, misfold both into amyloid structures that have a common macromolecular architecture and also into cytopathic oligomeric structures. Prion diseases provide a useful model system in this regard, since they are associated with *in vivo* protein misfolding into aggregates that can be oligomeric, fibrillar, neurotoxic and/or infectious, but the structures of such forms remain obscure [10].

**Figure 1:**
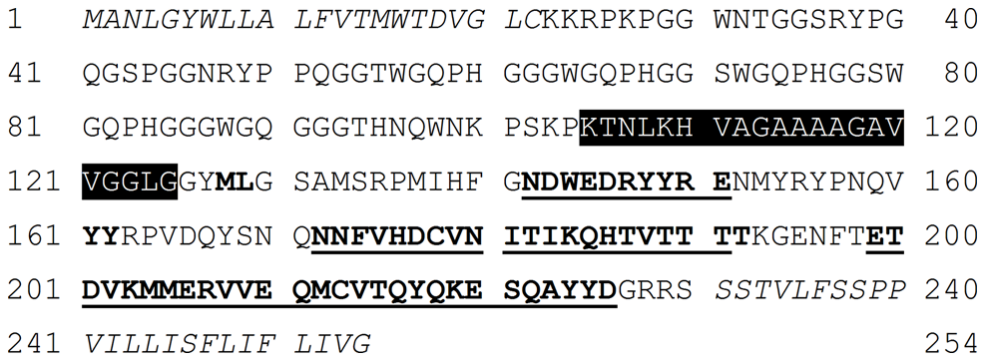
Sequence of the murine prion protein. The expressed protein has N- and C-terminal signal peptides (italics) that are removed during cellular processing. The N-terminal region is dynamically unstructured, whilst the C-terminal domain possesses globular structure, including two short sections of β-strand (bold text) with three α-helices (bold and underlined text). The hydrophobic peptide spanning residues 105–125 is highlighted in black with white text; the peptide spanning residues 108–121 is contained wholly within this peptide. Note, these peptides are homologous to human residues 106–126 and 109–122 and different numbering results from different lengths of the open reading frames. Throughout the rest of this paper the human numbering is used for consistency with other papers in the field.

Prion diseases are also known as transmissible spongiform encephalopathies and are associated with a conformational change of the cellular prion protein, PrP^C^. The native protein is structurally chimeric, composed of a partially-unstructured and flexible N-terminal region coupled to a globular C-terminal region by a short hydrophobic domain (Figure 1). The C-terminal region of PrP^C^ contains a 3-helix bundle with two short β-strands. During prion disease, the protein misfolds into a structure that is designated PrP^Sc^ and which contains substantially more β-sheet structure at the expense of α-helix [11], [12], [13]. PrP^Sc^ contains a protease-resistant core comprising residues ∼90 to the extreme C-terminus, indicating that the structural transition involves a short section of the N-terminal domain, the hydrophobic region and the entire C-terminal domain [14]. The atomic structure of PrP^Sc^ is unknown, but modeling studies, based on computer simulation with constraints derived from experiment, suggest that a β-helical fold may be present [15]. In this β-helical fibril model, the β-strands run perpendicular to the hypothetical fibrillar axis thereby satisfying the criteria of cross-β structure of amyloid fibrils. It is widely believed that the abnormal form of the prion protein acts as a template upon which naïve PrP^C^ monomers can misfold [16]. The results of many recent studies suggest that this template-directed, conformational change is a common facet of other protein misfolding diseases [17], [18], [19], [20], [21], [22], [23], [24], [25].

The central hydrophobic domain of the prion protein has a sequence that is highly conserved across all species studied to date [26], [27] and has properties that mimic those of the full molecule, such as the ability to misfold and aggregate into amyloid fibrils [28]. The core hydrophobic domain is composed of the residues 109–122 and was originally predicted to form an α-helical segment in the full length protein (hence was given the designation H1) [29]. A slightly longer version of this peptide, comprising residues 106–126, also misfolds and is associated with neurotoxicity [30]. Much research has focused on the conformations that can be adopted by the 109–122 and 106–126 peptides by use of biophysical techniques such as infrared spectroscopy, solution and solid phase NMR and ion mobility mass spectrometry, as well as a variety of molecular dynamics approaches [31], [32], [33], [34], [35], [36], [37], [38], [39], [40]. However, there has been a poor consensus from these studies, most likely because the peptides are conformationally flexible, the predominating conformation appears to depend strongly on solution conditions and many solution phase techniques for measuring secondary structure report only at the population average level. There have been corresponding studies of the mechanisms of neurotoxicity and these studies implicate deregulation of calcium ion homeostasis as a principal driver of cell death, possibly through oligomeric forms of the 109–122 and 106–126 peptides acting as integral-membrane ion channels [41], [42], [43], [44], [45], [46], [47].

In this study, all-atom, Monte Carlo simulations have been applied to study the conformations of the wildtype 109–122 and 106–126 peptides of murine PrP, as well as to equivalent peptides carrying a mutation associated with inherited, human prion disease. The simulations use a simplistic potential function that speeds computation and allows the study of multiple copies of peptides, thereby allowing models of aggregation to be explored. However, this also necessitates that the results be interpreted with care. Nevertheless, the energetically-favored conformation of all 4 monomeric peptides in implicit solvent was found to be essentially wholly α-helical, but cluster analysis also revealed that each peptide possess a small population of molecules in β-hairpin conformations. The disease-associated mutation increases the prevalence of β-hairpin forms of both 109–122 and 106–126 peptides, by increasing the energy of helical folds. Simulations of multiple copies of either 109–122 or 106–126 peptide chains in a periodic box show that transient dimers/trimers can form that are composed of molecules in β-hairpin conformation and that such assemblies can nucleate the formation of fibril-like aggregates composed of cross-β peptide structures. However, also evident during simulations are long-lasting, molecular assemblies, consistent with oligomers and composed of peptides in β-hairpin conformation. These data highlight β-hairpin structures as key mediators of the aggregation of prion peptides and provide a molecular basis for the assembly of such peptides into both oligomers and fibrils.

## Methods

### 2.1 Nomenclature

All simulations are based on peptide sequences derived from murine prion protein corresponding to residues 109–122 or 106–126. Mutated forms of both peptides were used and are given the designations 109–122 A_117_V and 106–126 A_117_V. In all peptides the N- and C-termini were derivatized with acetyl and amide groups respectively. Thus, the sequences of the peptides used are: 109–122, Acetyl-LKHVAGAAAAGAVV-NH_2_; 109–122 A_117_V, Acetyl-LKHVAGAAVAGAVV-NH_2_; 106–126, Acetyl-KTNLKHVAGAAAAGAVVGGLG-NH_2_; 106–126 A_117_V, Acetyl-KTNLKHVAGAAVAGAVVGGLG-NH_2_. Throughout this paper the human numbering is used for consistency with other papers in the field.

### 2.2 Molecular Simulations

All simulations were performed by use of the computer modeling software Profasi, (available at http://cbbp.thep.lu.se/activities/profasi/), the details of which have been previously published [48], [49]. Briefly, Profasi contains an implementation of the all-atom, implicit solvent force field developed by Irback and Mohanty to model the folding and aggregation of peptides ∼20 residues in length. Most bond lengths and angles as well as peptide torsion angles are fixed, such that each amino acid has the Ramachandran angles φ and ψ, as well as some sidechain torsional angles, as degrees of freedom. The interaction potential is composed of terms describing local interactions, excluded volume, hydrogen bonding and sidechain potentials and is described in detail elsewhere [48]. Conformational aspects of peptide folding are studied by Monte Carlo methods and each Monte Carlo step involves variable updates of 3 different types, the probability of each depending on the simulation temperature.

For simulations of monomers, the initial starting conformations were chosen randomly and the simulation software calculated a suitably sized box to contain the molecules during simulation. In the case of the 109–122 and 109–122 A_117_V peptides, this box had height, width and depth equal to 65.3 Å whilst for the longer 106–126 and 106–126 A_117_V peptides each dimension was 91.9 Å. For multi-molecular simulations, 20 copies of each respective molecule were used per simulation and each copy was assigned a random starting conformation. The 20 molecules were placed into cubes in which the height, width and depth dimensions were set to be equal to 100 Å for all peptides. The effective concentration of each peptide was therefore 33 mM, which equates to ∼43 mg/ml for the 109–122 and 109–122 A_117_V peptides and ∼63 mg/ml for the 106–126 and 106–126 A_117_V peptides. Monte Carlo simulations of the monomeric peptides were calculated using simulated tempering at 8 different temperatures, spaced equally between 277 K and 333 K (i.e. 277, 284, 292, 300, 308, 316, 324, 333 K). Note that these temperature designations are nominal, but since the computational model’s temperature scale has been previously calibrated against 20mer peptides then it is expected that they will be close to experimental values. Simulated tempering is similar to replica exchange or parallel tempering, in that temperature acts as a dynamic variable during the simulations, but in simulated tempering only one replica of each molecule is used. During the simulation the temperature can be altered to a neighboring temperature with predetermined probabilities depending, in part, on the total system energy. For simulations of each monomeric system, probabilities were determined iteratively by performing a series of short simulations and updating probabilities after each. This allowed the final simulations to sample all temperatures with roughly equal frequencies.

### 2.3 Analysis of Simulations

For simulations of monomeric structures, 1×10^8^ cycles were performed corresponding to ∼5.5×10^9^ or ∼8.5×10^9^ elementary Monte Carlo steps for the 109–122 and 106–126 peptides respectively. Coordinate files were written every 1,000 cycles yielding 100,000 structures per simulation. Structural and energetic data relating to each structure were output to populate histograms and this data was used for all graphical illustrations in this manuscript, except where specified. For more detailed structural analyses, every 20^th^ structure was written to separate pdb files, thereby yielding 5,000 structures per simulation, and these were analyzed for hydrogen bond frequencies and to assign structures to conformational clusters. Hydrogen bond frequencies were calculated using MolMol and hydrogen bonds were deemed to be present in a structure if the distance between donor and acceptor atoms was 2.4 Å or less and the angle of the donor-acceptor bond was not greater than 35°. Full lists of hydrogen bonds found are available on request.

Initial clustering of structures was performed using Gromacs (V4.6 [50]) using a cutoff of 2.5 Å for the 109–122 peptides and 3.5 Å for the 106–126 peptides. Clusters were compiled into larger ‘superclusters’ by means of a bespoke algorithm according to criteria outlined in supplementary information. Multi-molecular simulations were carried out at either 293 K or 303 K and were performed for 2×10^7^ cycles, corresponding to ∼2.4×10^10^ or ∼3.5×10^10^ elementary Monte Carlo steps. Coordinates of all atoms in the system were output every 500 cycles yielding 40,000 snapshots of the system over the course of each simulation. ‘Oligomerization’ of the peptide chains was monitored according to previously published criteria [51], [52]; briefly peptides were assumed to be part of an oligomeric assembly if they were connected to another chain by at least 3 hydrogen bonds, possessed at least 50% β-sheet structure and were oriented either parallel or antiparallel to each other to within 30°. For analysis and the production of videos of the simulations, every 100^th^ coordinate set was used. Structural representations and videos of multimolecular simulations were prepared by use of MolMol [53] or Visual Molecular Dynamics [54].

## Results

The central hydrophobic domain of PrP^C^ appears to be capable of conformational flexibility in solution, depending on the environment, and structures ranging from α-helix, through random coil to β-turn have previously been suggested. A simulated-tempering, Monte Carlo approach was used to probe the balance between these different structural forms for the 109–122 peptide as well as for a mutated version of the peptide in which alanine 117 was altered to a valine (A_117_V). This mutation occurs in humans and is associated with the familial prion disease Gerstmann-Sträussler-Scheinker syndrome [55]. The full, expressed sequence of the murine prion protein, with the central hydrophobic domain highlighted, is shown in figure 1.

### 3.1 The Wildtype 109–122 Peptide Preferentially Adopts α-helical Structure

In all work detailed in this manuscript, the all-atom, implicit solvent, molecular dynamics program Profasi has been used for Monte Carlo simulations of peptide structure. This software uses an implementation of a forcefield developed by Irback & Mohanty [48]; whilst this forcefield is a rather simplified approximation and may overstate electrostatic interactions, its speed allows studies that are not possible using higher resolution models. For instance, the software has previously been used to investigate differences in structure and aggregation propensity between different sequences of the Aβ peptide that accumulates during Alzheimer’s disease [52], [56], [57], [58], [59]. In the current study, the monomeric peptide homologous to murine PrP residues 109–122 was simulated over >10^9^ discrete Monte Carlo steps, using a simulated tempering approach in which different simulation temperatures were sampled with predetermined probabilities. Every 1,000 steps, a variety of information relating to the peptide sequence was output, including the total energy and the amount of secondary structure present.

**Figure 2:**
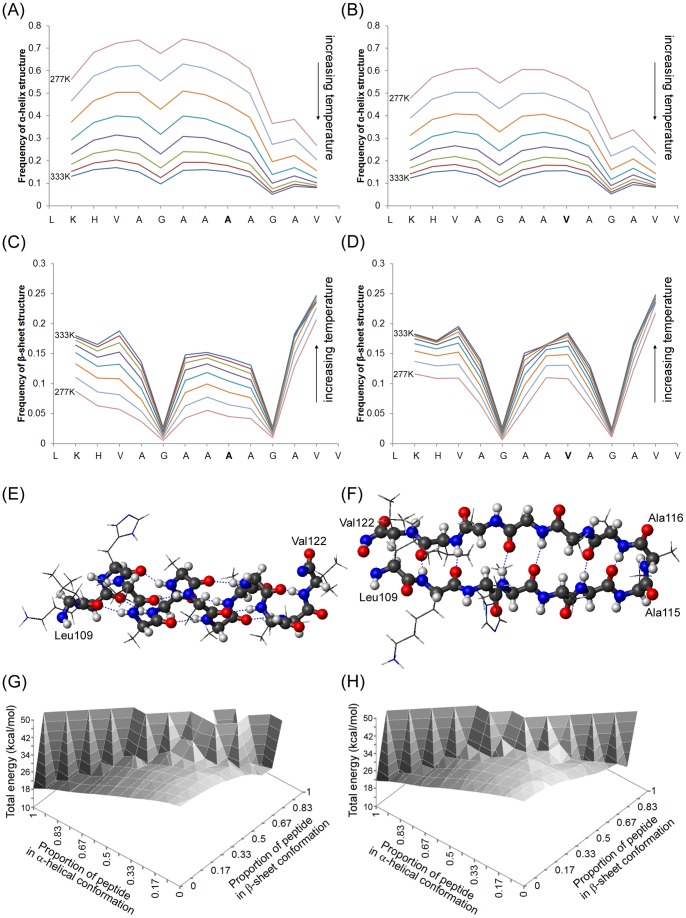
Analysis of Monte Carlo simulations of 109–122 and 109–122 A_117_V peptide monomers. (A) and (B) frequency of α-helical structure around each amino acid as a function of temperature for 109–122 and 109–122 A_117_V peptides respectively. Simulation temperatures are given in materials and methods and as the temperature is increased so the overall frequency of helical structure decreases. (C) and (D) frequency of β-sheet structure around each amino acid as a function of temperature for 109–122 and 109–122 A_117_V peptides respectively. As temperature increases the frequency of β-sheet also increases. (E) Schematic representation of the lowest energy conformation from the 3 replicate simulations of the wildtype 109–122 peptide. The backbone of the peptide is highlighted (carbon atoms grey, oxygen atoms red, nitrogen atoms blue) and the peptide is essentially 100% α-helical (F) schematic representation of the lowest energy conformer from the 3 replicate simulations of the wildtype 109–122 peptide in which β-sheet percentage is >50%. The peptide is in a β-hairpin conformation with a β-turn around residues Ala_115_ and Ala_116_. (G) and (H) total energy as a function of the proportion of helical structure and the proportion of β-sheet structure in 109–122 and 109–122 A_117_V mutant peptides respectively.

By use of the molecular potential implemented within Profasi, the wildtype 109–122 peptide is seen preferentially to adopt α-helical conformations. Figure 2(A) shows the frequency that each amino acid is in α-helical conformation as a function of simulation temperature whilst Figure 2(C) shows the frequency of β-sheet structure. Compared to the remainder of the peptide, the C-terminal region has a reduced propensity for helical structure, but, overall, there is an overriding preference for helical structure, particularly at lower simulation temperatures. The lowest simulation temperatures are associated with the lowest propensity for β-sheet structure and as the simulation temperature increases so does the frequency of β-sheet structure formation. However, it is notable that β-structure is uniformly absent from the two central glycine residues at all simulation temperatures.

The simulation of the 109–122 monomer was repeated 3 times with comparable results. Within each of the 3 repeats, the lowest energy conformer was ∼100% α-helical and the lowest energy conformer across all three replicate simulations is shown in Figure 2(E). Also evident were low energy states having high levels of β-sheet structure; these conformations predominately involved hairpin turns. Across all 3 simulations of the wildtype 109–122 structure, the lowest energy β-hairpin structure involves a β-turn around residues A_115_ and A_116_ and a schematic of this structure is shown in Figure 2(F). To probe the overall energetics of the 109–122 peptide, the average energy of the peptide was calculated as a function of the fraction of amino acids in α-helical and β-sheet structure. The 3-dimensional energy profile that results is shown in figure 2(G) and contains energy minima corresponding to high levels of α-helical structure or β-sheet structure respectively, separated by a saddle point corresponding to random structures. This suggests that structured conformers are energetically more stable than random conformations. Furthermore, Figure S1(A) in File S1 shows histograms depicting the distribution of peptide energies at different temperatures and shows that at low simulation temperatures, where many peptides have high helical structure, peptides possess a general lower energy than high temperatures at which random coil and sheet-containing structures predominate. If we focus only on structures derived from simulation temperatures of 284 or 292 K, the peptides segregate into those having high helical content and low energies and those having essentially zero helical content and higher energies (Figure S1 (B) in File S1). However, a small number of peptides having no helical content are seen to have low energies and these correlate to β-hairpin isoforms. Overall, the peptide 109–122 preferentially forms helical conformations but can also form β-hairpin structures having low energies, whilst random coil structures are associated with higher energies.

### 3.2 The Disease-associated Mutation A_117_V Increases β-sheet Structure in the 109–122 Peptide by Reducing the Thermodynamic Stability of Helical Forms

Three replicate simulations of the 109–122 peptide carrying the disease-associated mutation A_117_V were carried out, in order to determine the effect that this mutation may have on the conformational dynamics. Similarly to the wildtype peptide, the lowest energy conformation of the 109–122 A_117_V peptide, across 3 replicate simulations, is also ∼100% α-helix, however there are differences in the overall propensity for helical structure compared to the wildtype peptide. For the mutated peptide, the frequencies of α-helical and β-sheet structure at each amino acid, as a function of simulation temperature, are shown in Figures 2(B) and 2(D) respectively. Compared to the wildtype 109–122 peptide (Figures 2(A) and 2(C)), the mutated peptide has a reduced propensity to form α-helical structure at low temperatures, coupled with a higher propensity for β-sheet structure at the same temperatures. The reason for this alteration in conformational dynamics is a reduction in the thermodynamic stability of helical forms of the mutated peptide relative to the wildtype peptide. Figure 2(H) shows the energy landscape as a function of α-helical and β-sheet structure for the 109–122 A_117_V peptide; this shows a similar overall profile to the energy landscape for the wildtype peptide (Figure 2(G)), but the average energies of helical forms of the 109–122 A_117_V structure is increased. This is in contrast to other conformations, particularly β-sheet-containing structures, which have similar energies to their wildtype counterparts. A destabilization of helical forms can also be seen by comparing energy distributions at low simulation temperatures (Figure S1 (A) and (C) in File S1), which have higher energies relative to similar simulations of the wildtype peptide. The 3-dimensional histograms shown in Figure S1 (B) and (D) in File S1 also show that, for peptide structures at simulation temperatures of 284 or 292 K, there is a shift in the population of peptides from high helical content to essentially zero helical content as a result of the mutation. Thus, overall the A_117_V mutation destabilizes α-helical folds and increases the proportion of other conformations in the total population.

### 3.3 The A_117_V Mutation Increases the Percentage of Conformers of the 109–122 Peptide having β-hairpin Structure

Based on the increased preference for β-sheet formation and the postulated ability of the 109–122 peptide to form β-hairpin structures [40], [60], [61], it was hypothesized that the A_117_V mutation in the 109–122 peptide quantitatively promotes the formation of β-hairpin structures at the expense of helix. To test this, 5,000 structures were extracted at regular intervals from each replicate simulation, yielding 15,000 independent structures for each of the 109–122 and 109–122 A_117_V peptides respectively. These structures were first clustered, based on root-mean-squared deviations in atomic coordinates, but this yielded many clusters of <5 structures each. The initial clusters were further amalgamated based on the presence of particular secondary structural motifs to produce 14 structural ‘superclusters’ associated with specific structural elements and a 15^th^ that accounted for all other peptide structures (i.e. random coil). The details of this procedure are given in the supplementary materials along with the theoretical definitions of each of the superclusters and example structures assigned to each (Table S1 in File S1).

The 14 structured superclusters of the 109–122 peptide included several associated with α-helical structure as well as superclusters involving β-hairpin structure. The superclusters were further consolidated to produce 5 structural families corresponding to structures that (i) were largely α-helical (ii) were β-hairpin in structure with a turn comprising two or more of the residues 113–116 (iii) were β-hairpin in structure with a turn comprising two or more of the residues 117–119 (iv) were double β-hairpins with turns at both residues 113–116 and 117–119 or (v) were random coil. The percentage of structures present in each of these top level families are shown in Table 1 for simulations of both the 109–122 and 109–122 A_117_V peptides. These data show clearly that the A_117_V mutation results in an increase in the number of molecules in β-hairpin conformation where the turn is around residues 113–116 and there is an almost 2 fold greater number of molecules having a double β-hairpin structure comprising two turns. There are less structures having α-helical structure in the simulation of the 109–122 A_117_V peptide relative to its wildtype counterpart and overall these data suggest that the mutated peptide does indeed lose helical structure but gains certain β-hairpin structures. To allow more detailed comparisons of the results of the mutation, Table S2 in File S1 shows the number of structures assigned to each of the 15 superclusters, along with the fold change that results.

**Table 1:** Summary table of the average percentage of structures in different structural superclusters formed by the 109–122 and 109–122 A_117_Vpeptides during simulations.

Structural family description	109–122 (%)	109–122 A_117_V (%)	Fold change
Family associated with α-helical structure	45.07	38.05	0.84
β-hairpin structures with β-turns around residues 113–116^a^	2.84	7.09	2.50
β-hairpin structures with β-turns around residues 117–119^b^	2.38	2.07	0.87
Double β-hairpin	1.15	2.15	1.88
Random coil	48.56	50.63	1.04

a comprises hairpin structures with β-turns at residues 113–114, 114–115 or 115–116.

b comprises hairpin structures with β-turns at residues 117–118, or 118–119. Table S2 in File S1 lists all 15 superclusters individually along with the average percentage of structures within each cluster.

β-hairpin structures divide into several different subsets depending on the type of turn that links the antiparallel β-strands of such structures. β-hairpin structures of the 109–122 and 109–122 A_117_V peptide structures were analyzed to attempt to determine whether particular types of turn predominated. Some β-hairpin structures possessed α-turns, but the majority of structures had β-turns, which comprise two residues in the turn and hydrogen bonds linking residues *i* and *i*+3. Within this population, a preference was found for β-turns of the II or II’ type, however, in many structures the backbone Ramachandran angles deviated substantially from the ideal angles for any of the recognized β-turns [62]. This is probably a result of the inflexibility of bond lengths and certain bond angles in the computer model, as well as reflecting the dynamic nature of the conformation of the peptide during Monte Carlo simulations at higher temperatures. Nevertheless, II and II’ β-turns are preferred in β-hairpin structures and henceforth the generic term ‘β-turns’ will be used to describe all turns linking antiparallel β-strands in β-hairpin structures.

To confirm that the apparent increase in β-hairpin structures were not artifacts of the clustering methodology, a second method of analysis was used. The 5,000 structures from each simulation (3 replicate simulations each of 109–122 and 109–122 A_117_V) were analyzed for frequently occurring hydrogen bonds. From such data, it is possible to determine the change in percentage occupancy of each hydrogen bond in the 109–122 peptide that results from the A_117_V mutation. Those hydrogen bonds that show a 2 fold change in percentage occupancy (or greater) are listed in Table 2. Hydrogen bonds showing the largest changes tend to be between main-chain atoms of residues that are some distance apart in the linear structure. In other words, they are associated with bonding between N- and C-terminal domains in a hairpin structure. Table 2 also lists the approximate location of the turn in hairpin peptides that would account for the hydrogen bonding observed; for many of the hydrogen bonds undergoing the largest fold change as a result of the A_117_V mutation, the turn would be around residues 113–115. Thus, from the simulations undertaken of the 109–122 and 109–122 A_117_V peptides, there is unequivocal evidence that that the presence of the A_117_V mutation causes increased prevalence of β-hairpin structure in the 109–122 peptide at the expense of α-helical folds.

**Table 2:** Hydrogen bonds showing changes in occupancy >2 fold between 109–122 and 109–122 A_117_V peptide simulations.

		Average hydrogen bond occupancy^b^				
H-bond donor^a^	H-bond acceptor^a^	109–122 (%)	109–122 A_117_V (%)	Fold-change		β-turn residues
K_110_	A_118_	0.09	0.57	6.07		113–115
A_118_	H_111_	1.02	3.75	3.67		113–115
H_111_	A_118_	0.55	2.00	3.67		113–115
G_119_	V_122_	0.31	1.11	3.53		n/a
V_122_	A/V_117_	1.03	2.97	2.90		119–120
A_113_	A_116_	1.58	4.51	2.86		114–115
K_110_	A/V_117_	0.40	0.94	2.35		113–114
A_120_	L_109_	0.30	0.70	2.33		114–115
A/V_117_	K_110_	0.95	2.2	2.32		113–114
A/V_117_	L_109_	0.47	1.04	2.23		112–114

a in all cases the hydrogen bond donor atoms were amide nitrogen atoms and acceptors were carbonyl oxygen atoms of the specified residues.

b hydrogen bond frequencies quoted are the average occupancies of the 3 discrete simulations for each peptide; each simulation contributed 5000 structures for hydrogen bond determination.

### 3.4 A Longer Peptide, Encompassing Residues 106–126, Replicates the Behavior of the 109–122 Peptide

**Figure 3:**
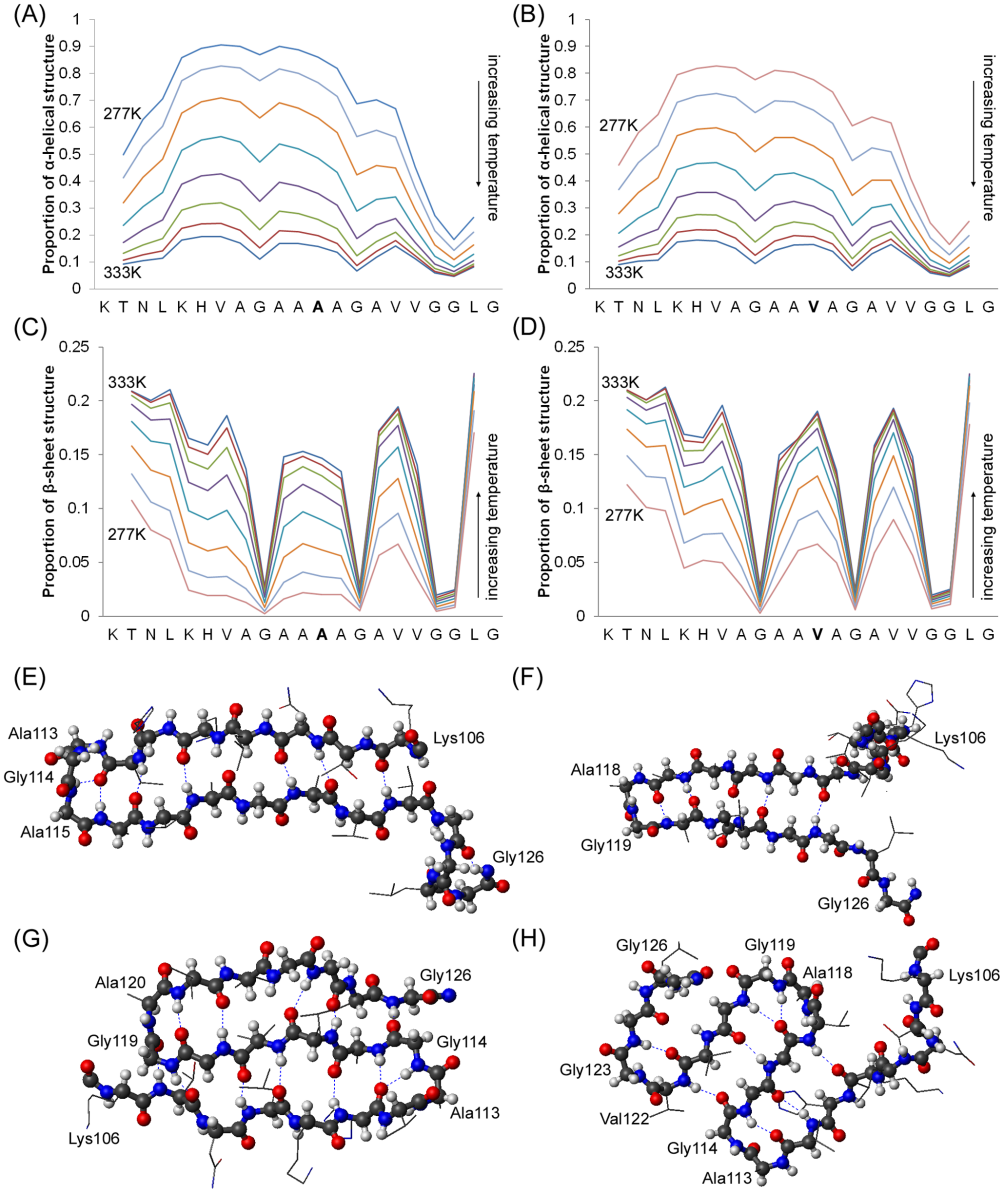
Analysis of Monte Carlo simulations of 106–126 and 106–126 A_117_V peptide monomers. (A) and (B) frequency of α-helical structure around each amino acid as a function of temperature for 106–126 and 106–126 A_117_V peptides respectively. Simulation temperatures are given in materials and methods and as the temperature is increased so the overall frequency of helical structure decreases. (C) and (D) frequency of β-sheet structure around each amino acid as a function of temperature for 106–126 and 106–126 A_117_V peptides respectively. As temperature increases the frequency of β-sheet also increases. (E) Schematic representation of the 106–126 A_117_V peptide as a β-hairpin with a β-turn located around residues 113–115. The backbone of the peptide is highlighted (carbon atoms grey, oxygen atoms red, nitrogen atoms blue) (F) Schematic representation of the 106–126 A_117_V peptide as a β-hairpin with a β-turn around residues 118–119. (G) Schematic representation of the 106–126 A_117_V peptide as a double β-hairpin with β-turns around residues 113–114 and 119–120 (H) Schematic representation of the 106–126 A_117_V peptide as a triple β-hairpin conformation with β-turns around residues 113–114, 118–119 and 122–123. In all hairpin structures the β-turn incorporates one of the glycine residues of peptide 106–126.

Much research in the prion field has focused on the structural, fibrillogenic and neurotoxic properties of a longer version of the 109–122 peptide, comprising residues 106–126. To confirm results obtained for the 109–122 peptide, the longer 106–126 peptide and its mutated equivalent (106–126 A_117_V) were subjected to 3 replicate Monte Carlo simulations in Profasi. The results are comparable to those generated from the 109–122 peptides in that the peptide spanning residues 106–126 is preferentially α-helical and the majority of low energy conformations have >90% helical content. Apart from the extreme C-terminal amino acids, GGLG, all residues show high frequencies of helix formation at low temperatures, with helical content decreasing as temperature is increased (Figure 3(A)). Levels of β-sheet, per residue, are low at low temperatures and plateau as the temperature is increased (Figure 3(C)). Similarly to the 109–122 A_117_V peptide, the mutated 106–126 A_117_V peptide has reduced levels of α-helix (Figure 3(B)) and higher levels of β-sheet (Figure 3(D)) in the central portion of the peptide relative to the wildtype 106–126 peptide at the same temperatures. The presence of the valine in the 106–126 A_117_V peptide again results in a shift to higher energies at the lowest temperature sampled (compare Figure S2 panels (A) and (B) in File S1), whilst the higher temperatures, associated with random coil structure, show little differences in energy. The energy landscapes for the 106–126 and 106–126 A_117_V peptides also have minima occurring at high levels of α-helix and of β-sheet structure (Figure S2 (C) and (D) in File S1) and the mutation shifts the minimum associated with high levels of helix to a higher energy. At intermediate temperatures, the A_117_V mutation causes a decrease in the number of structures populating helical states in favor of high energy, unstructured forms and low energy β-sheet containing forms (Figure S3 in File S1).

To quantify the balance between populations of the different structural conformers, 15,000 structures from the wildtype 106–126 simulations and an equivalent number from the 106–126 A_117_V peptide simulations were clustered into 29 superclusters (Tables S3 & S4 in File S1) and further consolidated to 6 structural families, as outlined in Table 3. This includes different β-hairpin forms with turns at 3 different locations (e.g. Figure 3(E) and (F)), double hairpin (Figure 3(G)) and triple hairpin forms (Figure 3(H)). As with the 109–122 peptide, the presence of the A_117_V mutation in the 106–126 peptide caused increases in the propensity for formation of β-hairpin forms at the expense of helical conformers. This is also reflected in the change in hydrogen bond occupancies; those hydrogen bonds that show the most dramatic changes in occupancy as a result of the A_117_V mutation (Table 4) are consistent with formation of β-turns around residues 113–115 and 119–121. These are the same locations in which β-turns are found in the shorter 109–122 and 109–122 A_117_V peptides. In summary, simulations of the monomeric wildtype 106–126 and mutated 106–126 A_117_V peptides replicate those of the shorter 109–122 and 109–122 A_117_V peptides, indicating that β-hairpin forms represent a small subset of structures, but that the prevalence of these forms increases after A_117_V mutation.

**Table 3:** Average percentage of structures in superclusters formed by the 106–126 and 106–126 A_117_V peptides during simulations.

Supercluster description	106–126 (%)	106–126 A_117_V (%)	Fold change
Cluster associated with α-helical structure	50.47	44.47	0.88
Single β-hairpin with β-turns around residues 112–117^a^	1.19	2.75	2.32
Single β-hairpin with β-turns around residues 118–120^b^	1.55	1.91	1.24
Double β-hairpin with β-turns at residues 112–116 and/or 118–120 and/or 122–124^c^	0.77	2.44	3.16
Triple β-hairpin	0.02	0.23	11.67
Random coil	46.01	48.19	1.05

a Comprises hairpin structures with β-turns at residues 112–114, 113–114, 114–115, 115–116 or 116–117.

b comprises hairpin structures with β-turns at residues 118–119, 118–120 or 119–120.

c comprises double hairpins that have turns at two of the locations detailed under a and b above or β-turns at residues 122–124.

**Table 4:** Hydrogen bonds changing in occupancy >2 fold as a result of the A_117_V mutation in simulations of the 106–126 and 106–126 A_117_V peptides.

		Average hydrogen bond occupancy b				
H-bond donor^a^	H-bond acceptor^a^	106–126 (%)	106–126 A_117_V (%)	Fold-change	β-turn residues	
N_108_	A_120_	0.08	0.41	5.17	113–115	
L_109_	A/V_117_	0.15	0.73	4.95	112–114	
G_119_	T_107_	0.12	0.57	4.78	112–114	
A_120_	N_108_	0.13	0.59	4.40	113–115	
A_118_	H_111_	0.57	2.51	4.38	114–115	
K_110_	A_118_	0.15	0.63	4.32	113–115	
H_111_	A_118_	0.39	1.63	4.21	114–115	
A/V_117_	L_109_	0.25	0.99	4.03	112–114	
A_113_	A_116_	0.84	3.15	3.75	114–115	
N_108_	G_119_	0.08	0.29	3.58	113–114	
A_120_	L_109_	0.15	0.53	3.43	114–115	
G_123_	A_116_	0.70	2.39	3.42	119–120	
A_116_	G_123_	0.25	0.81	3.27	119–120	
A_118_	V_121_	0.86	2.77	3.22	119–120	
V_122_	A/V_117_	1.06	3.38	3.19	119–120	
K_110_	A/V_117_	0.46	1.39	3.03	113–114	
G_123_	A_116_	0.30	0.85	2.84	119–120	
L_125_	G_114_	0.13	0.36	2.84	119–120	
G_119_	V_122_	0.76	2.05	2.70	120–121	
G_119_	N_108_	0.37	0.96	2.62	113–114	
A/V_117_	K_110_	0.89	2.31	2.58	113–114	
A_116_	G_124_	0.15	0.39	2.57	119–121	
H_111_	A_115_	0.43	1.03	2.42	112–114	
V_112_	A_115_	1.34	3.02	2.25	113–114	

a in all cases the hydrogen bond donors were amide nitrogen atoms and acceptors were carbonyl oxygen atoms of the particular residues.

b hydrogen bond frequencies quoted are the average occupancies of the 3 discrete simulations for each peptide; each simulation contributed 5000 structures for hydrogen bond determination.

### 3.5 Multi-chain Simulations Indicate that the 109–122 Peptide can form Stable Fibrils Composed of Extended, Anti-parallel β-strands in a Cross-β Architecture

The Profasi software provides the facility for simulating the behavior of multiple peptide chains contained within a virtual cube of defined dimensions. This allows the study of how peptides may interact to form multi-molecular assemblies. To simulate aggregation of the 109–122 peptide in implicit solution, 20 copies of this peptide were placed within a virtual cube in which the vertices each measured 100 Å. 3 replicate simulations were performed at 293 K and a further 3 were performed at 303 K. These temperatures were chosen based on the results of simulations of the peptide monomer; at these temperatures there should be sufficient structural flexibility to populate many different conformations over time, as well as sufficient translational motion to allow peptide-peptide interactions to be sampled. Simulations were performed for ∼2.3×10^10^ elementary Monte Carlo steps and the total energy, percent of α-helical and β-sheet structure as well as the extent of ‘oligomerization’ (defined in materials and methods) were monitored at regular intervals. Short videos of all multi-molecular simulations undertaken during this work are available as supplementary materials (Video S1 through Video S12).

**Figure 4:**
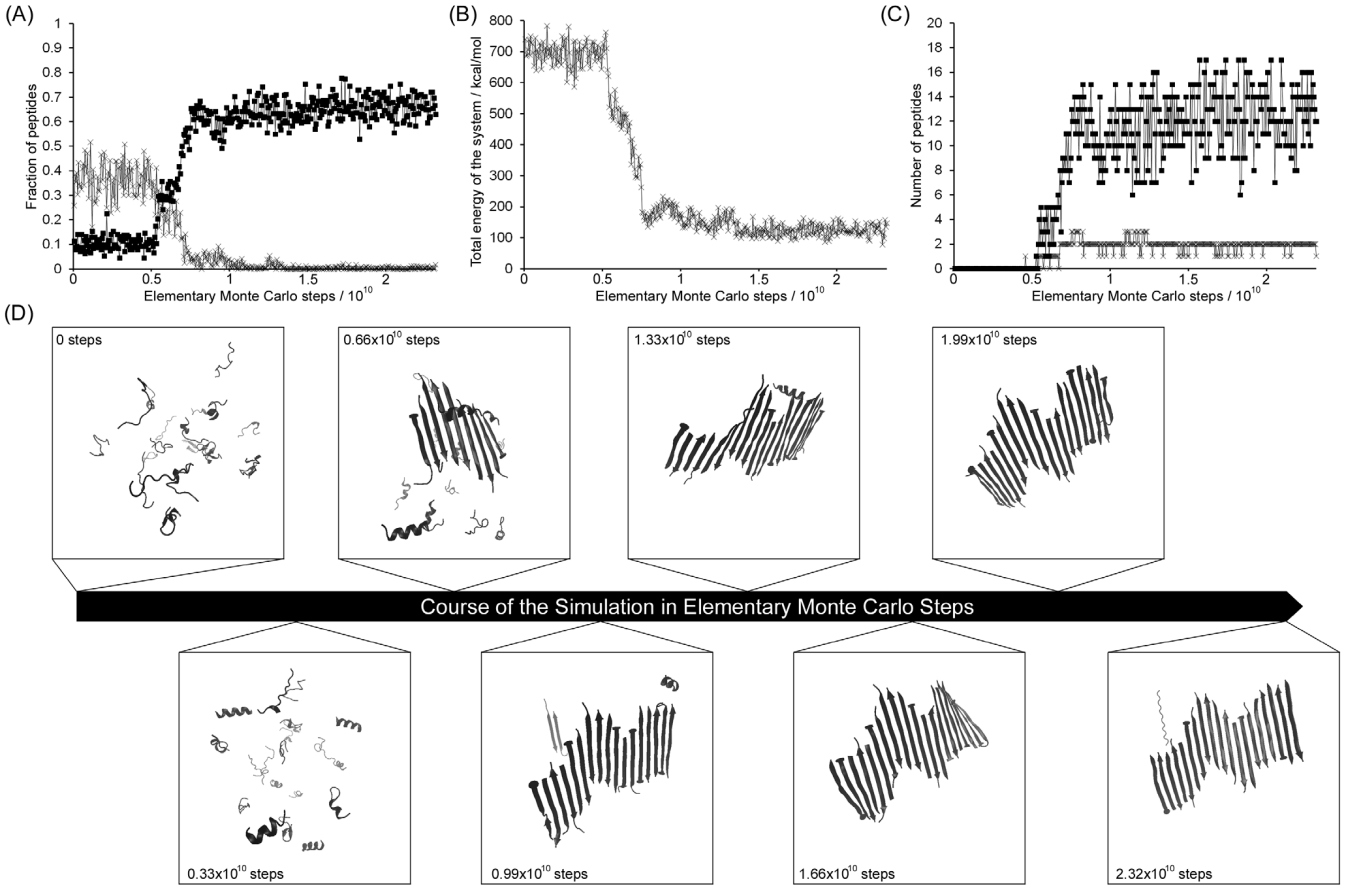
In some multi-peptide simulations of the 109–122 peptide, large, multi-molecular assemblies are formed. The data in this figure relates to replicate 1 at a simulation temperature of 293(A) fraction of peptide having α-helical structure (×) or β-sheet structure (▪) as a function of Monte Carlo steps. (B) The total energy of the system as a function of Monte Carlo steps. (C) the amount of peptides in parallel β-sheets (×) or in anti-parallel β-sheets (▪) as a function of Monte Carlo steps. (D) Ribbon representations of snapshots of the system at regular points throughout the simulation, as indicated.

Of the 6 multichain simulations of the 109–122 peptide, 3 showed no signs of aggregation and the population of peptides at the end of the run contained disperse monomers in a range of conformations (Table S5 in File S1). By contrast, the other 3 simulations showed a dramatic association of peptides into ordered, β-sheet-containing structures. An example is shown in Figure 4. Figure 4(A) shows the fraction of the peptides that are in α-helical or β-sheet structure. From ∼0.5×10^10^ steps there is an increase in β-sheet structure at the expense of helical structure. There is a concomitant reduction in the total energy of the system (Figure 4(B)) and an increase in the number of oligomerized peptides (Figure 4(C)). Snapshots of the peptide population over the course of the simulation (Figure 4(D)) show clearly that individual peptides adopt β-strand conformations and associate into a single, large, multi-stranded β-sheet; in other simulations more than one peptide assembly formed during the simulations. Once these structures formed they were stable for the remainder of the simulations and the final morphology (Figure 4(D) at 2.32×10^10^ steps) is similar to a proteinaceous fibril, where β-strands run perpendicular to the axis of the fibril.

The algorithm that monitors the association of peptides distinguishes between β-strands that are parallel from those that are antiparallel. In all of the simulations of the 109–122 peptide that result in aggregation, there is an overriding preference for antiparallel β-sheet formation (Figure 4(C)). Peptides are not all ‘in-register’, hence some seem to be off-set from the fibril axis thereby producing deviations from an ideal fibrillar structure. However, once they began to form, peptides associated into the fibrillar structures rapidly and these structures were then stable for the course of the simulations. This produced an association phase that shows the classic sigmoidal geometry of fibril formation when monitored by the conformational transition (Figure 4(A)), total energy of the system (Figure 4(B)) and peptide associations (Figure 4(C)). In addition, the absence of fibril formation in some simulations, coupled with the rapid, sigmoidal association phase in those simulations that do result in fibrils, is consistent with a stochastic nucleation event preceding fibril formation, hence the pre-association phase was carefully analyzed.

### 3.6 Prior to Fibrillization, Small Multimers of the 109–122 Peptide form Transiently and are Composed Partly of Peptides in β-hairpin Conformation

The pre-association phase of multi-chain simulations of the 109–122 peptide is characterized by multiple inter-molecular interactions. For the system shown in Figure 4, the steps immediately prior to and spanning the initial association phase were dissected in detail; 4,000 snapshots of the system covering the period from 0.42–0.65×10^10^ steps were analyzed for hydrogen bonds between the different chains and some of the data that resulted is shown in Figure S4 in File S1. Hydrogen bonds between two specific chains were often relatively transient, often lasting only 1 frame or so (equivalent to 6×10^5^ Monte Carlo steps). However, in some cases, interactions were evident that persisted over multiple frames (representing up to ∼5×10^7^ elementary Monte Carlo steps) as evidenced by many sequential frames in which hydrogen bonds numbered >2–3. These interactions are consistent with the formation of small oligomers (predominately dimers or trimers) and could be detected by quantifying hydrogen bonds formed only between two specific chains. For example, Figure S4(A) in File S1 shows all hydrogen bonds formed between backbone atoms of chain A and any other backbone atoms in the system. These data contain a sequential stretch of several frames containing >2 hydrogen bonds at around 0.5×10^10^ steps. This is caused by a two-way association with chain O, since a peak in the number of hydrogen bonds specifically between these two chains coincides with the prolonged interactions of chain A with the rest of the system. Likewise, chain C has an extended sequence of frames containing >3 hydrogen bonds around steps 0.49×10^10^ steps resulting from an interaction with chain P (Figure S4 panel (C) in File S1); chain Q undergoes 2 interactions with chains D and N (Figure S4 panel (E) in File S1).

During simulations of the monomeric 109–122 peptide, the most prevalent defined structures were α-helices and β-hairpins (Table 1). Reasoning that hairpin structure may be important for the formation of transiently-stable multimers, the frequency of hairpin structures was analyzed over the same 4,000 snapshots of pre- and mid-association phases of the fibrillizing multichain system. In general, any given chain formed β-hairpin structure regularly, but this structure existed only for single frames at a time (Figure S4 panels (B), (D) and (F) in File S1). However, when small oligomers were detected, at least one of the chains involved in the aggregates existed in β-hairpin structure for a substantially greater number of frames. For example, Figure S4(B) in File S1 shows that chain O exists as a β-hairpin for almost the entire time that it is associated with chain A and for some of this time chain A is also in β-hairpin conformation. Chain P is in β-hairpin conformation during its association with chain C (Figure S4(D) in File S1) and chain N forms a β-hairpin structure during association with chain Q (Figure S4(F) in File S1). Metastable multimers can form in which neither partner is in hairpin conformation (e.g. chains Q & D in Figure S4(F) in File S1), but such complexes do not persist for more than a few sequential frames hence are not readily detected. In other words, β-hairpin formation by at least one partner in an oligomer appears important to produce a prolonged association.

**Figure 5:**
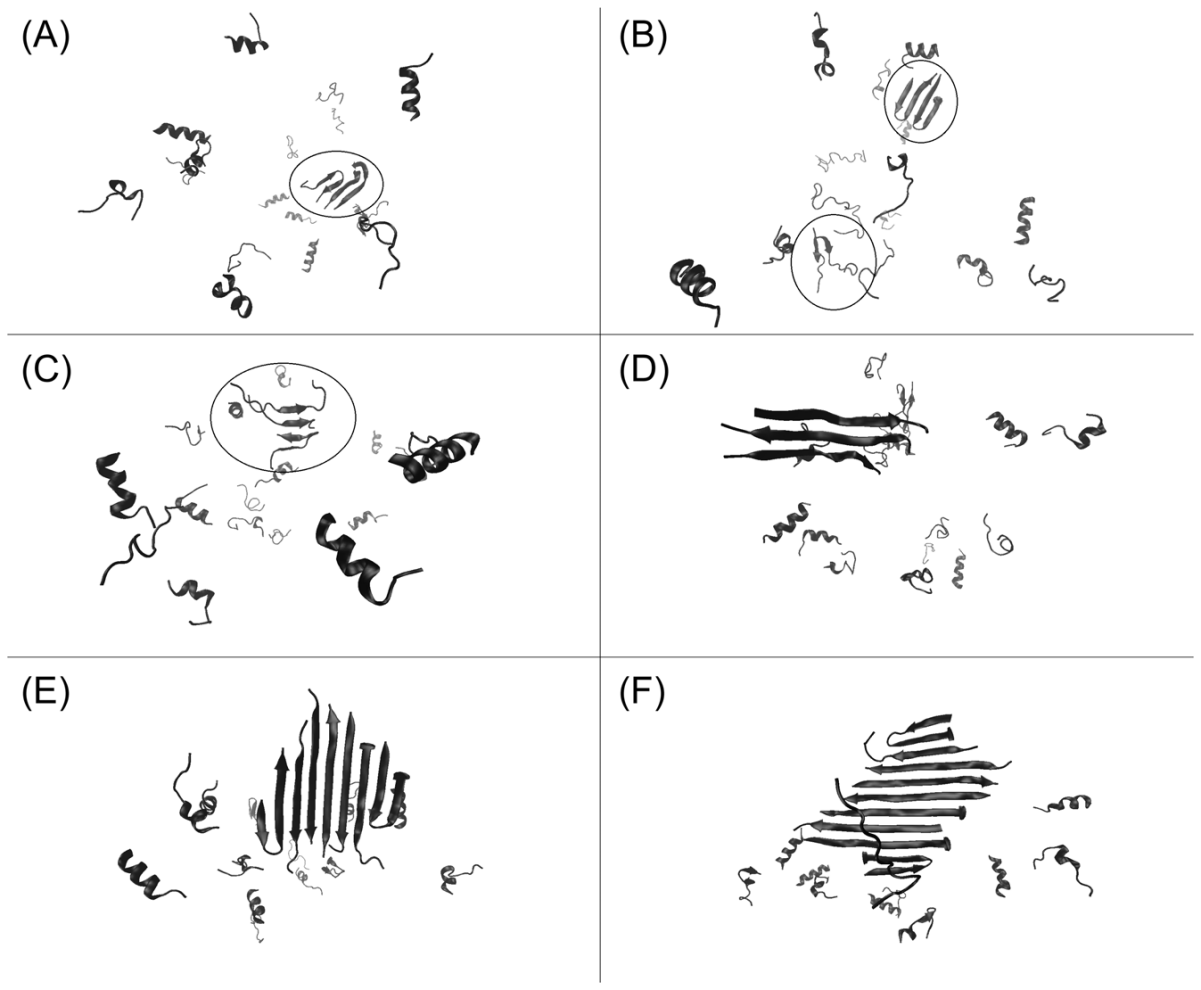
Molecular assemblies that form during the pre- and mid-association phase of fibrillization of the 109–122 peptide. (A–C) Ribbon representations of transiently stable multimers formed prior to nucleation of fibrillization. Dimers and trimers form that are often composed, partly, of peptides in β-hairpin conformation. (D) Ribbon representation of the trimer that represents the nucleus of fibril formation and is composed of peptides that have formed linear β-strands. (E–F) Ribbon representations of the growing fibril shortly after nucleation occurred. Peptides that have added to the ends of the growing fibril are often in β-hairpin conformation for a large number of Monte Carlo steps and inhibit addition of more peptides until the β-hairpin peptides open to form extended β-strands.

Several examples of small multimers that form during the pre-fibrillization stage, prior to 0.54×10^10^ steps, are highlighted in Figure 5 within the ellipses. These include two trimers in which one of the partners adopts β-hairpin conformation (Figure 5 (A) & (C)), as well as a dimer containing two β-hairpins and a dimer containing no β-hairpins (both Figure 5(B)), demonstrating the different generic structures of oligomers that can form and then dissipate. By contrast, Figure 5(D) shows a trimer composed of peptides in elongated β-strand conformations. This multimer represents the nucleus of fibril formation for this simulation to which other chains subsequently add; the nucleus forms initially as a conformationally-flexible dimer to which the third chain adds, thereby stabilizing the hydrogen bond network. The nucleus exists as a trimer composed of extended β-strands for ∼10^8^ Monte Carlo steps before more chains begin to add to the ends of the growing β-sheet. The major difference between this trimer and others that form only transiently is that the peptide chains form extended β-strands, rather than β-hairpins, allowing backbone-to-backbone hydrogen bonds down the entire length of each peptide chain. Thus, once small oligomers have formed, linearization of peptide structure is an important step in fibrillogenic nucleus formation.

### 3.7 Hairpin Structures may be Required for Peptide Docking to the Fibril, but then such Peptides at the Termini of Fibrils Impede Elongation Until they form Extended Structures

β-hairpin structures appear important during the pre-fibrillization phase of growth to promote long-lived, intermolecular interactions. β-hairpin conformations are also preferred substrates to add to the ends of growing fibrils, since they already contain sections of β-sheet. However, once peptides have added to the end of the fibrils, β-hairpin structure inhibits further addition of monomers. Figure 5 (E) & (F) show snapshots of the same 109–122 system discussed above shortly after nucleation of the fibril (at 0.553 and 0.649×10^10^ steps respectively). Peptides in β-hairpin conformation can clearly be seen at each end of the fibril. These snapshots are taken from the region of the simulation encompassing steps 0.5–0.7×10^10^ and it can be seen in Figure 4 (A–C) as well as Figure S4 (A) & (C) in File S1 that there exists a pronounced plateau in the elongation phase of fibril growth. The presence of the β-hairpin structures at each end of the fibril prevent further addition of monomer units until these peptides have undergone conformational change to produce extended β-strands. Thus, overall the pre-association phase is characterized by the formation and dissipation of small oligomers composed partly of peptides in β-hairpin conformation, whilst such hairpins must form linear β-strands to allow fibril-like structures to form. A detailed video of the pre- and mid-association phase of this system, which demonstrates all the behavior discussed in the previous two sections, is available as supplementary materials (Video S21).

### 3.8 The 106–126 Peptide forms Different Molecular Assemblies Composed of β-hairpins, Extended β-strands or Mixtures of these Structures

**Figure 6:**
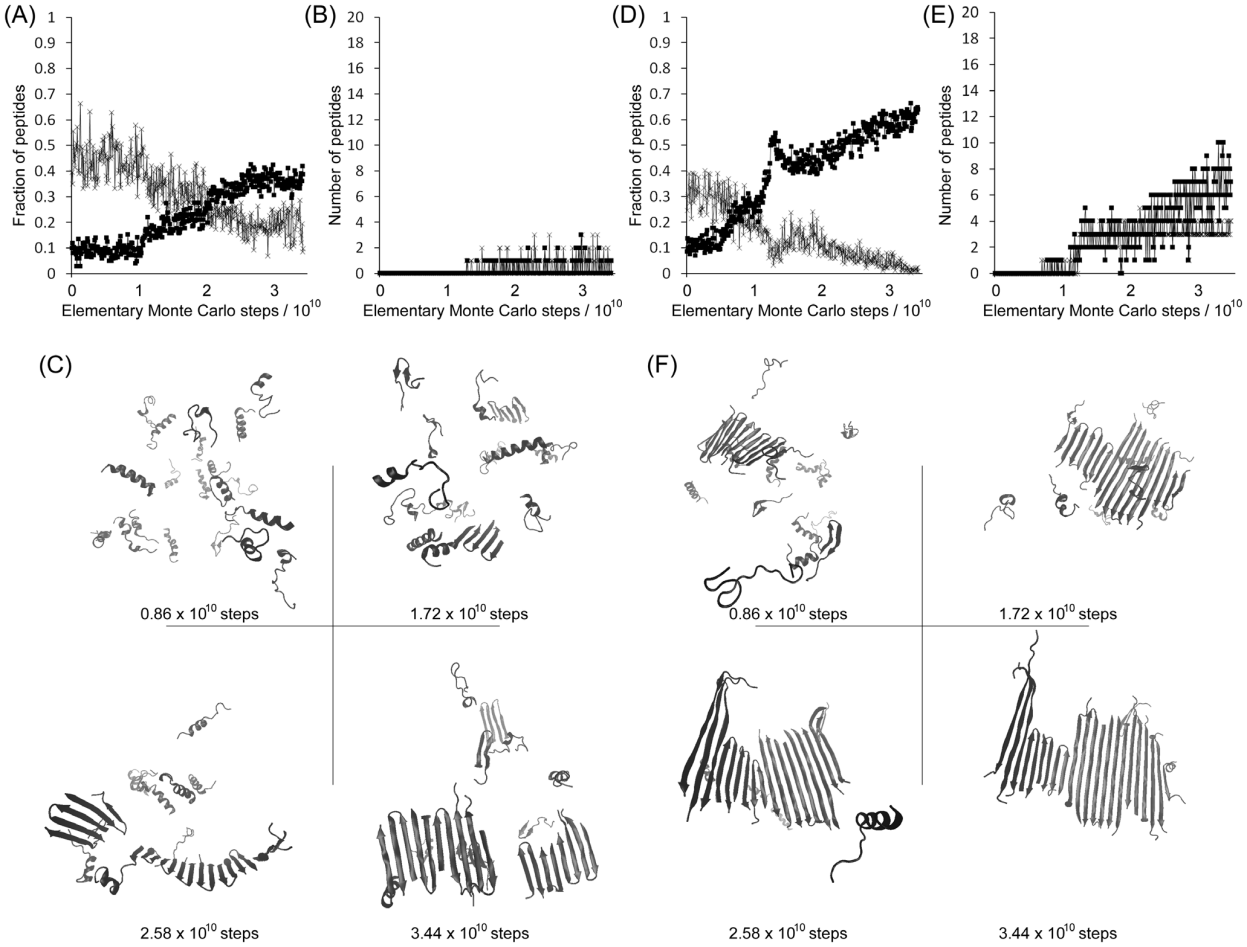
Structurally-different multimeric assemblies of the 106–126 peptide can form during simulations. Panels (A–C) relate to replicate 1 at 293 K, whilst panels (D–F) relate to replicate 1 at 303 K. (A) and (D) show the fraction of peptide that possesses α-helical structure (×)or β-sheet structure (▪) as a function of Monte Carlo steps. (B) and (E) show the extent of oligomerization of peptide chains into parallel (×) or anti-parallel (▪) β-sheets as a function of Monte Carlo steps. (C) and (F) show ribbon representations of the state of the peptide ensemble at regular time points during each simulation. In panel (C), assemblies composed of peptides in β-hairpin conformation form roughly halfway through the simulation period. In panel (F), a nucleus of peptides in β-hairpin conformation form early during the simulation and, eventually, peptides being to form a fibril-like structure composed of β-strands.

Multichain simulations of the 106–126 peptide have also been carried out at both 293 and 303 K. Results, including final ensemble morphologies, are shown in Table S5 in File S1 and videos of all simulations are available as supplementary materials (Videos S13 through S20). Unlike the shorter 109–122 peptide, the 106–126 peptide appears to associate more readily and all multichain simulations resulted in the formation of multimeric assemblies. However, the morphologies of these assemblies varied between different simulations. The results of two such simulations are depicted in Figure 6. The simulations result in a gradual increase in β-sheet at the expense of α-helical structure (Figure 6(A) and (D)) and a similarly gradual increase in chains associating into multimers (Figure 6 (B) and (E)). Structurally-different multimeric assemblies can form during the simulations, which, in common with the 109–122 peptide, are associated with either β-hairpin conformations or with linear β-strands. Figure 6(C) shows snapshots from a simulation in which small oligomers formed that were composed of β-hairpin conformations. These oligomers persisted for more Monte Carlo steps than those formed by the 109–122 peptide, presumably because the longer 106–126 peptide allows for greater numbers of hydrogen bonds between peptides. In some cases the oligomers dissipated after being assembled for several billion Monte Carlo steps.

By contrast, Figure 6(F) shows snapshots from a system in which a large ‘fibrillar’ assembly forms composed principally of peptides in extended β-strand conformations. The nucleus for formation of this fibril is a small oligomer comprising 3–4 peptides in β-hairpin conformation, suggesting that oligomers of this type may be capable of initiating fibril formation if there is sufficient conformational overlap between the monomer units in each molecular aggregate. Indeed, other simulations of the 106–126 peptide show further evidence of ‘mixed’ assemblies comprising peptides having either β-hairpin sections or extended β-strands (Table S5 in File S1). The single replicate simulation in which extensive fibrillization occurs shows a greater number of anti-parallel β-strands compared to parallel, but overall it is difficult to say whether parallel or anti-parallel β-strands are preferred. In summary, the 106–126 peptide can associate into structures composed of β-hairpins, extended β-strands or mixtures of both conformations. The contribution of each of these structures in the growing aggregate appears down to both thermodynamic and kinetic factors; if β-hairpin peptide can open to form linear β-strands before addition of the next peptide to the growing aggregate then fibril-like assemblies will predominate but if peptides add rapidly to the growing aggregate then β-hairpin-containing, oligomer-like structures will be preferred. This, in turn, depends on the stability of β-hairpin structures.

### 3.9 Multichain Simulations Indicate that the A_117_V Mutation may Reduce the Propensity of Peptides to Fibrillize Whilst Aiding Oligomer Formation

Finally, multichain simulations of both the 109–122 A_117_V and 106–126 A_117_V peptides were carried out. In simulations of the corresponding monomers, the A_117_V mutation enhanced formation of β-hairpin structure in both peptides. For the 109–122 A_117_V peptide, 3 of the 6 simulations resulted in fibril-like assemblies at the end of the simulation period, a similar level of fibrillization to the wildtype 109–122 peptide (Table S5 in File S1). The 106–126 A_117_V peptide formed assemblies of molecules in β-hairpin conformation in all 4 of the simulations performed. Therefore, the mutation does not appear to affect the propensity for the peptides to coalesce into multimers. However, the presence of the A_117_V mutation in the 109–122 peptide appeared to reduce the rate of fibril elongation. Table S5 in File S1 shows plots of the extent of oligomerization of the peptide population over time for all multimolecular simulations undertaken in this work; compared to plots for the wildtype peptides, those for the mutated 109–122 peptides show a reduced rate of structural change as well as a reduced rate of oligomerization after nucleation. This is consistent with β-hairpin-mediated nucleation of oligomers being equally efficient for both wildtype and mutated 109–122 peptides, whilst the reorganization of peptides to form extended β-strands is more efficient for the wildtype peptide than for the mutated peptide.

Incorporating the A_117_V mutation into the longer 106–126 peptide affects neither the initial nucleation of oligomerization nor the subsequent growth phase, presumably because the multimers formed in these simulations are composed largely of peptides in β-hairpin conformation. Since the A_117_V mutation enhances β-hairpin formation in the simulations of monomers of this peptide, this result is not unexpected; indeed one may predict that β-hairpin-containing oligomeric assemblies of this peptide may be favored over fibril-like assemblies containing extended β-sheet structures as is seen in the shorter peptide. Substantially more repeat simulations may allow significant differences in the course of multimerization to be elucidated. Thus, overall it appears that the A_117_V mutation slows growth of fibril-like assemblies whilst not affecting the formation or growth of β-hairpin-containing oligomers.

## Discussion

Throughout this study, simulations were performed by use of software that implements a simplified, course-grained force field, which has been optimized to describe the folding of small peptides and proteins. Whilst the approximations used greatly increase the speed of calculation, they may also result in incorrect treatment of certain atomic interactions. The results must therefore be treated with some caution and interpreted, where possible, in the context of *in vitro* research; fortunately there exists several experimental studies of prion peptide folding. In the current simulations, the energetically most stable structure of both 109–122 and 106–126 peptides was found to be essentially 100% α-helix. However, cluster analysis of 15,000 structures output from simulations at regular intervals demonstrated a small but significant population of conformers having β-hairpin structures, whilst the predominating conformation (statistically) was random coil. These results seem in reasonable agreement with experiment; prion peptides can form helical conformations in mixtures of water and trifluoroethanol, whilst in pure water the helical conformations are replaced by higher levels of β-structure, possibly because the peptides are already aggregating. It is possible that the force field used in these simulations overestimates the prevalence of ‘structured’ conformations, because hydrogen bonding energies are artificially high, but it is also possible that the balance of conformational states predicted by simulation represent the true ensemble adopted by the peptide in solution.

It should be noted that the formation of β-hairpins in prion peptide has previously been suggested explicitly and implicitly in a range of other studies, further serving to validate the current simulations. In this work, β-hairpin structures varied in relation to the number of turns – single, double and triple hairpin forms of peptides were found – as well as the location of the turns, but the majority of turns were of the β type and incorporated one of the glycine residues of the peptides. Glycine residues are known to be favored in β-turns as a result of the flexibility of the backbone torsional angles [63] whilst alanine residues, that comprise the central portion of the PrP peptides, are predominately helix-favoring but are also capable of turn formation [64]. Substitution of alanine 117 for valine, a mutation that is associated with inherited prion disease in humans [55] and which leads to enhanced toxicity of peptide aggregates *in vitro* [65], energetically destabilized helical folds and increased the proportion of peptides in β-hairpin conformation. This effect is in line with the known preference for valine residues to be in β-strands instead of α-helix [66]. Depending on the force field and solvent conditions used, computer simulations have shown the potential for β-hairpin formation in the peptides used in the current study [37], [60], [61], [67], [68], [69]. Furthermore, studies of synthetic peptides have found evidence for β-hairpin formation *in vitro* in the 106–126 peptide [40].

An increasing body of literature implicates β-hairpin formation as an essential step in misfolding of a range of peptides associated with amyloid formation such as Aβ [70], [71], amylin [72], [73], [74] and the polyglutamine region of amyloidogenic huntingtin [75]. Thus, there have been several suggestions that β-hairpin peptide conformations may represent key intermediates that promote the nucleation of amyloid fibril formation. The current study supports this hypothesis and outlines the molecular mechanisms of β-hairpin mediated aggregation in unprecedented detail. In multichain simulations of aggregation, both 109–122 and 106–126 prion peptides formed transiently-stable, oligomeric assemblies that comprised at least one peptide in β-hairpin conformation. For the 109–122 peptide, these oligomers often persisted for only a short amount of simulation time, whilst for the longer peptide they appeared more stable. However, both peptides also formed assemblies with architecture more akin to that found in amyloid fibrils, with characteristic cross-β structure and composed of peptides in linear β-strand conformation. For the 109–122 peptide the structural transition from β-hairpin-containing oligomer to fibril occurred rapidly and completely, whilst the longer peptide formed molecular assemblies composed of mixtures of β-hairpin and linear β-strands. Whilst the β-hairpin-driven mechanism of aggregation may be an artifact of the force field used for these simulations (since it may overestimate the strength of backbone-backbone hydrogen bonds), it should be noted that the same force field has been used in other studies of aggregation in other amyloid-prone peptide systems and has predicted somewhat different mechanisms for initial coalescence of monomers [51], [52], [58], [59].

The formation of ‘oligomers’ consisting of β-hairpin structure and fibrils consisting of linear β-strands is consistent with a wealth of data on the role of hairpin formation in fibrillogenic peptides. To undergo the transition from β-hairpin to linear β-strand, the turn within hairpin peptides needs to straighten. This process can be seen clearly in videos of the current simulations, available as supplementary information, after hairpin peptides add to the ends of the growing fibrils. But if the β-hairpin structures are not easily able to ‘open up’ to form extended β-strands then this will have the effect of slowing fibrillization kinetics. Stabilization of β-hairpin structure has continually been found to inhibit the formation of amyloid fibrils whilst promoting oligomer formation in Aβ [76], [77], [78], [79], [80], polyglutamine peptides [81] and the short amyloidogenic peptide KFFE [82]. Moreover, peptides designed to form β-hairpin structure can interfere with amylin and α-synuclein fibrillization [83] and with Aβ aggregation [84]. Conversely, conditions that reduce β-hairpin stability also increase the rate of fibril formation [57], [85]. Consistent with these reports, the A_117_V substitution in the 109–122 peptide enhanced the population of β-hairpin peptides and appeared to slow the formation of fibril-like growth once nucleation had occurred. There have also been reports that the same substitution enhanced the formation of unstructured forms of the peptide [86]. Also within the prion protein hydrophobic region, substitution of the two central glycine residues reduces fibrillization, presumably by reducing backbone flexibility. It is around these glycine residues that the majority of β-turns occur. Collectively, these studies appear to point to the importance of β-hairpin structure in nucleating oligomer formation, but once oligomers have formed the stable hairpin structures are less capable of maturing to form *bona fide* amyloid fibrils. It is therefore interesting that disease-associated mutations in other proteins also appear to shift the balance of aggregation from fibrils to more toxic oligomers [87], [88], [89].

It is pertinent to ask how generic – and physiologically relevant – the current molecular description of oligomers and fibrils of the prion hydrophobic domain is likely to be. Recent computational studies of Aβ aggregation have yielded similar mechanisms of nucleated growth, in which β-hairpin-containing (or amorphous) oligomers form rapidly, but fibril-like growth subsequently requires the formation of extended β-strands to result in a fibril-inducing seed [51], [90], [91]. This model of fibrillization is reinforced by experimental evidence of several unrelated proteins that also form precursor aggregates, which then reorganize into fibril-competent structures [92], [93], [94]. For the prion hydrophobic domain, there has also been experimental evidence for reorganization of peptide chains both after initial nucleation of fibril formation and while the fibril is growing. This reorganization involves reptation – a realignment of β-strands to produce a regular alignment [95] – which in the case of prion peptide 109–122 may ultimately result in the alignment of residue 117 in peptide β-strands organised in an anti-parallel architecture [33], [96], [97]. Anti-parallel strands with residue 117 in alignment would theoretically produce a core hydrophobic region encompassing the palindromic sequence VAGAAAAGAV. This core region has previously been suggested to be a key determinate of both fibril formation and neurotoxicity of the 106–126 peptide [98], [99] and has also been shown experimentally to be the most protected from hydrogen/deuterium exchange [100]. This core region is critically important for β-hairpin formation in the current study and this region may also form β-hairpins in the context of a substantially longer prion fragment [101], [102]. Thus, the current data are consistent with both theoretical and experimental observations of peptide misfolding and probably represent the initial phases of fibril growth only, such that if the simulations present herein were extended then they may result in alignment of A/V_117_ in antiparallel strands by molecular rearrangements within fibrils. It is also worth noting that whilst many studies of peptide aggregation have found evidence for parallel architecture in β-strands in fibrils, there have been a number of studies that identify anti-parallel architecture as being important for fibril formation of specific peptides, including several studies on prion peptides [38], [94] and Aβ [103], [104].

Finally, one key limitation of the Profasi software used for these simulations is the move set; single-body rigid body translations are used and when more than one molecule interacts then the assembly is almost immobile in space. This effect can be visualized in all videos where aggregation occurs, since the molecular assembly appears spatially fixed once it has formed. In reality, small movements are possible, but the reduced lateral movement prevents the coalescence of multiple nuclei that may form concomitantly. This will also prevent two larger fibrillar assemblies coming together to form double layer sheets of the kind found recently in amyloid-forming peptides, called steric zippers. Steric zippers have been found in crystal structures of several amyloid-forming peptides [38], [105], [106] and involve the interdigitation of amino acid side chains between pairs of β-strands, thereby forming hydrophobic interfaces from which water molecules are excluded. Such structures are attractive explanations for fibril conformations of proteins, since they can rationalize amyloid stability as well as structural polymorphism seen in amyloid aggregates [107], although it remains unclear whether dry, steric zipper interfaces are formed only during *in vitro* crystallization [108]. In the current study both β-hairpin-containing oligomers and extended conformation fibrils have faces that could form steric zippers and it would be interesting to extend the simulations to investigate whether coalescence of individual fibers occurs. This could be achieved if the simulation time is dramatically extended, if an upgraded multi-molecular translation scheme is used or if the preformed fibrils are moved to higher resolution molecular dynamics packages for simulation.

In conclusion, this study has revealed a key molecular mechanism of association of peptides derived from the hydrophobic region of the prion protein. β-hairpin conformations in individual monomers is a critical step in both initial oligomer formation as well as monomer addition to nucleated fibrils. The data also suggest that conditions that stabilize β-hairpin structures may slow fibril formation, but may also increase formation of oligomers, the species that have repeatedly been reported to be cytotoxic Nevertheless, β-hairpin formation in the hydrophobic region of prion proteins could represent an important therapeutic target to prevent protein misfolding during prion disease pathogenesis.

## Supplementary Information

Supplementary information available including: Detailed methodology for generation of superclusters of structures; Full lists of number of structures in each supercluster for simulations of 4 peptide monomers in this work; supplementary figures, as outlined in the text; Short videos of the 20 multi-chain systems generated in this work plus a longer video showing the molecular detail of the association of the 109–122 peptide into fibrils.

## Supporting Information

File S1 **Compiled supplementary information including (i) supplementary methods (ii) Table S1– Definitions of 109–122 superclusters (iii) Table S2– Populations of superclusters for the 109–122 and 109–122 A_117_V peptide simulations (iv) Table S3– Definitions of 106–126 superclusters (v) Table S4– Populations of superclusters for the 106–126 and 106–126 A_117_V peptide simulations (vi) Figure S1– Energy histograms for the 109–122 peptide and its A_117_V mutant (vii) Figure S2– energy histograms and total energy profiles of the 106–126 peptide and its A_117_V mutant (viii) Figure S3 - 2D histogram of energy verses α-helical content for the wildtype 106–126 peptide and its A_117_V mutant (ix) Table S5– Summary of all multi-chain simulations performed during this work (x) Figure S4 - Detailed plots of the pre- and mid-association phase from simulation of 20 copies of the 109–122 peptide at 293 K (replicate 1).**
(PDF)

Video S1 **Multi-molecular simulation of the peptide 109–122 at a temperature of 293 K, replicate 1.**
(WMV)

Video S2 **Multi-molecular simulation of the peptide 109–122 at a temperature of 293 K, replicate 2.**
(WMV)

Video S3 **Multi-molecular simulation of the peptide 109–122 at a temperature of 293 K,.replicate 3**
(WMV)

Video S4 **Multi-molecular simulation of the peptide 109–122 at a temperature of 303 K, replicate 1.**
(WMV)

Video S5 **Multi-molecular simulation of the peptide 109–122 at a temperature of 303 K, replicate 2.**
(WMV)

Video S6 **Multi-molecular simulation of the peptide 109–122 at a temperature of 303 K, replicate 3.**
(WMV)

Video S7 **Multi-molecular simulation of the peptide 109–122 A_117_V at a temperature of 293 K, replicate 1.**
(WMV)

Video S8 **Multi-molecular simulation of the peptide 109–122 A_117_V at a temperature of 293 K, replicate 2.**
(WMV)

Video S9 **Multi-molecular simulation of the peptide 109–122 A_117_V at a temperature of 293 K, replicate 3.**
(WMV)

Video S10 **Multi-molecular simulation of the peptide 109–122 A_117_V at a temperature of 303 K, replicate 1.**
(WMV)

Video S11 **Multi-molecular simulation of the peptide 109–122 A_117_V at a temperature of 303 K, replicate 2.**
(WMV)

Video S12 **Multi-molecular simulation of the peptide 109–122 A_117_V at a temperature of 303 K, replicate 3.**
(WMV)

Video S13 **Multi-molecular simulation of the peptide 106–126 at a temperature of 293 K, replicate 1.**
(WMV)

Video S14 **Multi-molecular simulation of the peptide 106–126 at a temperature of 293 K, replicate 2.**
(WMV)

Video S15 **Multi-molecular simulation of the peptide 106–126 at a temperature of 303 K, replicate 1.**
(WMV)

Video S16 **Multi-molecular simulation of the peptide 106–126 at a temperature of 303 K, replicate 2.**
(WMV)

Video S17 **Multi-molecular simulation of the peptide 106–126 A_117_V at a temperature of 293 K, replicate 1.**
(WMV)

Video S18 **Multi-molecular simulation of the peptide 106–126 A_117_V at a temperature of 293 K, replicate 2.**
(WMV)

Video S19 **Multi-molecular simulation of the peptide 106–126 A_117_V at a temperature of 303 K, replicate 1.**
(WMV)

Video S20 **Multi-molecular simulation of the peptide 106–126 A_117_V at a temperature of 303 K, replicate 2.**
(WMV)

Video S21 **Detail of the peptide association phase from the Multi-molecular simulation of the peptide 109–122 at a temperature of 293 K, replicate 1.**
(WMV)
