# Supplementary material for: β-Hairpin-Mediated Formation of Structurally Distinct Multimers of Neurotoxic Prion Peptides
Source: PLoS One. 2014 Jan 31;9(1):e87354. doi: 10.1371/journal.pone.0087354 (PMC3909104; doi:10.1371/journal.pone.0087354)
Supplement: File S1 — Compiled supplementary information including (i) supplementary methods (ii) Table S1– Definitions of 109–122 superclusters (iii) Table S2– Populations of superclusters for the 109–122 and 109–122 A117V peptide simulations (iv) Table S3– Definitions of 106–126 superclusters (v) Table S4– Populations of superclusters for the 106–126 and 106–126 A117V peptide simulations (vi) Figure S1– Energy histograms for the 109–122 peptide and its A117V mutant (vii) Figure S2– energy histograms and total energy profiles of the 106–126 peptide and its A117V mutant (viii) Figure S3 - 2D histogram of energy verses α-helical content for the wildtype 106–126 peptide and its A117V mutant (ix) Table S5– Summary of all multi-chain simulations performed during this work (x) Figure S4 - Detailed plots of the pre- and mid-association phase from simulation of 20 copies of the 109–122 peptide at 293 K (replicate 1). (PDF) [file pone.0087354.s001.pdf]

“β-hairpin-mediated formation of structurally distinct multimers of neurotoxic prion peptides”

Andrew C. Gill

Written Supplementary Materials

Supplementary Methods:

Analysis methodology for creation of superclusters of peptide structure.

For each simulation of monomeric peptides, 5,000 individual structures were output at regular intervals during the course of the simulation. Taking each simulation in isolation, initial clustering of structures was performed by Gromacs, based on root-mean-squared deviations of α-carbon atoms. Clustering used a cutoff of 2.5 Å for the 109-122 peptides and 3.5 Å cutoff for 106-126 peptides. Clustering was performed by means of the Gromos method, which uses the algorithm as described in Daura et al. 1999. In this algorithm, the number of “neighbours” of a given structure is calculated according to the defined cut-off, and the structure with largest number of neighbours is assigned as cluster 1; this structure and all its neighbours are eliminated from the pool and the process is repeated for remaining structures in the pool until all structures are assigned to clusters. From the starting pool of 5,000 structures there were typically >1,000 clusters formed and, in many cases, clusters had only one structure present. Hence, the clusters were further merged according to the presence of particular secondary structural elements, which were empirically defined by visual inspection of the top 100 clusters of each peptide simulation. Merging of Gromacs clusters were done, by use of bespoke clustering algorithms, as follows:

For each of the 5,000 peptide structures in each replicate simulation, the per-residue secondary structure was calculated according to DSSP by means of the Gromacs software and the frequency of secondary structure per residue was averaged for all structures within each cluster. This produced an average level of helix, sheet, turn and coil per residue for each cluster. Specific, objective rules were created to define the structural requirements for particular structural super-clusters. These definitions and the corresponding rules to allow superclustering of the 109-122 peptides are shown below in Supplementary Table S1, whilst a description of the superclusters of the 106-126 peptide are given in Supplementary Table S3. For the 109-122 peptide and its mutated form, the molecule was split into 3 parts for determination of helical content. This was arbitrarily defined as the N-terminal region (residues 110-113), the central region (residues 114-117) and the C-terminal region (118-121). For the 106-126 peptide and its mutated form, the 3 regions were defined as N-terminal (residues 107-112), central region (113-119) and C-terminal region (120-125). Each rule was applied sequentially, in the order outlined in Supplementary Tables S2 and S4 below (for 109-122 and 106-126 peptides respectively), and once a cluster had been defined to a particular supercluster group, it was not reassigned, even if a cluster could theoretically fit into more than one group (i.e. a cluster may have N-terminal helix but also a C-terminal hairpin turn, in which case it is put in the first supercluster to which it applies based on the rules). In practice, few clusters fit rules for more than one supercluster.

Daura, X.; Gademann, K.; Jaun, B.; Seebach, D.; van Gunsteren, W. F.; Mark, A. E., Peptide folding: When simulation meets experiment. *Angewandte Chemie-International Edition* **1999,** 38, (1-2), 236-240.

Supplementary Table S1 – Definitions of 109-122 superclusters:

Cluster	Cluster Description	Rule	Example structure
1	Predominately helical	N-terminal domain > 50% helix<br>Central domain > 60% helix<br>C-terminal domain > 50% helix	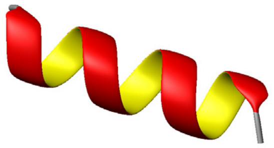
2	N-terminal and central domains helical, C-terminal domain not helical	N-terminal domain > 50% helix<br>Central domain > 60% helix<br>C-terminal domain < 50% helix	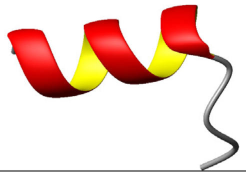
3	C-terminal and central domains helical, N-terminal domain not helical	N-terminal domain < 50% helix<br>Central domain > 60% helix<br>C-terminal domain > 50% helix	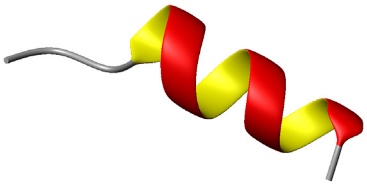
4	β-hairpin with a turn at residues 6 & 7	Res 6 & 7 have average of > 60 % turn<br>Res 5 & 8 have average of < 40% turn<br>Res 4, 5, 8 & 9 have average of > 50% sheet<br>Res 10 & 11 have average of < 50% turn<br>Res 9, 10 & 11 have average of < 50% turn	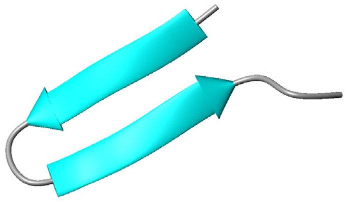
5	Double β-hairpin with turns at residues 6 & 7 and 10 & 11	Res 6 & 7 have > 60 % turn on average<br>Res 5 & 8 have < 40% turn on average<br>Res 4, 5, 8 & 9 have > 50% sheet on average<br>Res 10 & 11 have > 60 % turn on average<br>Res 9 & 12 have < 40% turn on average<br>Res 8, 9, 12 & 13 have > 50% sheet on average	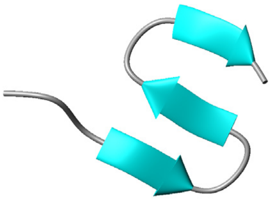
6	β-hairpin with a turn at residues 5 & 6	Res 5 & 6 have > 60 % turn on average<br>Res 4 & 7 have < 40% turn on average<br>Res 3, 4, 7 & 8 have > 50% sheet on average<br>Res 10 & 11 have < 50% turn on average<br>Res 9, 10 & 11 have < 50% turn on average	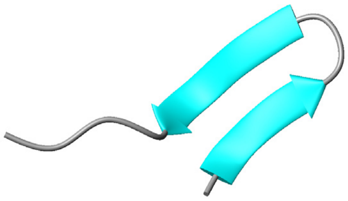
7	Double β-hairpin with turns at residues 5 & 6 and 10 & 11	Res 5 & 6 have > 60 % turn on average<br>Res 4 & 7 have < 40% turn on average<br>Res 3, 4, 7 & 8 have > 50% sheet on average<br>Res 10 & 11 have > 60 % turn on average<br>Res 9 & 12 have < 40% turn on average<br>Res 8, 9, 12 & 13 have > 50% sheet on average	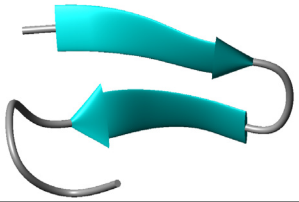
8	Double β-hairpin with turns at residues 5 & 6 and 9, 10 & 11	Res 5 & 6 have > 60 % turn on average<br>Res 4 & 7 have < 40% turn on average<br>Res 3, 4, 7 & 8 have > 50% sheet on average<br>Res 9, 10 & 11 have > 60 % turn on average<br>Res 8 & 12 have < 40% turn on average<br>Res 7, 8, 12 & 13 have > 50% sheet on average	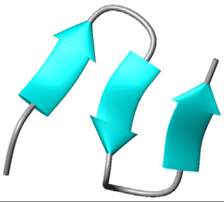
9	β-hairpin with a turn at residues 10 & 11	Res 10 & 11 have > 60 % turn on average<br>Res 9 & 12 have < 40% turn on average<br>Res 8, 9, 12 & 13 have > 50% sheet on average<br>Res 5 & 6 have < 50 % turn on average<br>Res 6 & 7 have < 50 % turn on average<br>Res 7 & 8 have < 50 % turn on average	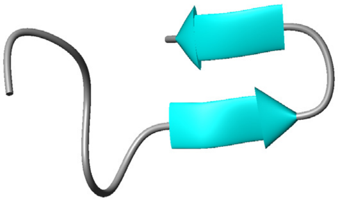

10	β-hairpin with a turn at residues 9, 10 & 11	Res 9, 10 & 11 have > 60 % turn on average<br>Res 8 & 12 have < 40% turn on average<br>Res 7, 8, 12 & 13 have > 50% sheet on average<br>Res 5 & 6 have < 50 % turn on average<br>Res 6 & 7 have < 50 % turn on average<br>Res 7 & 8 have < 50 % turn on average	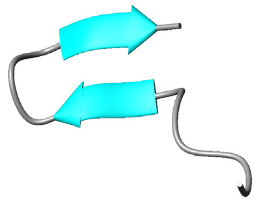
11	β-hairpin with a turn at residues 7 & 8	Res 7 & 8 have > 60 % turn on average<br>Res 6 & 9 have < 40% turn on average<br>Res 5, 6, 9 & 10 have > 50% sheet on average<br>Res 10 & 11 have < 50% turn on average<br>Res 9, 10 & 11 have < 50% turn on average	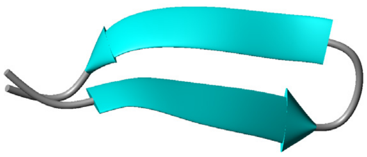
12	Central region helical but N- and C-terminal regions not helical	N-terminal domain < 50% helix<br>Central domain > 60% helix<br>C-terminal domain < 50% helix	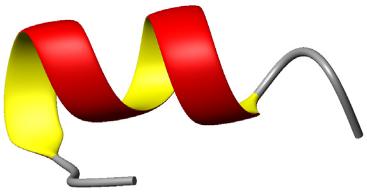
13	N-terminal region helical but central and C-terminal region not helical	N-terminal domain > 50% helix<br>Central domain < 60% helix<br>C-terminal domain < 50% helix	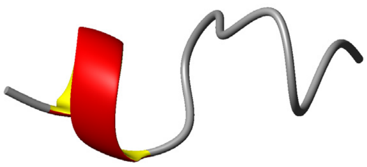
14	C-terminal region helical but central and N-terminal region not helical	N-terminal domain < 50% helix<br>Central domain < 60% helix<br>C-terminal domain > 50% helix	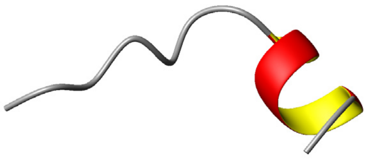
15	Unstructured or other	Does not conform to any of the rules defining the above superclusters	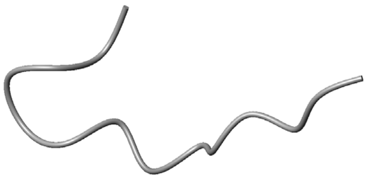

Supplementary Table S2 – Populations of superclusters for the 109-122 and 109-122 $A_{117}V$ peptide simulations

Rule order	Cluster (as per Supplementary Table S1)	109-122 (%)	109-122 $A_{117}V$ (%)	Change resulting from $A_{117}V$ mutation
1	Whole peptide predominately helical	18.97	16.23	0.86
2	Helical N-terminus and central region	10.29	6.41	0.62
3	Helical C-terminus and central region	1.73	2.03	1.17
13	N-terminal helical, rest random coil	9.19	7.88	0.86
14	C-terminal helical, rest random coil	1.41	1.72	1.22
12	Central region helical, termini random coil	3.79	3.87	1.02
6	β-hairpin, turn at residues 113-114	1.18	2.29	1.94
4	β-hairpin, turn at residues 114-115	1.07	3.38	3.15
11	β-hairpin, turn at residues 115-116	0.55	0.61	1.10
10	β-hairpin, turn at residues 117-119	0.63	0.75	1.18
9	β-hairpin, turn at residues 118-119	1.89	1.63	0.87
8	Double β-hairpin, turns at 113-114 and 117-119	0.51	0.86	1.70
7	Double β-hairpin, turns at 113-114 and 118-119	0.02	0.01	0.67
5	Double β-hairpin, turns at 114-115 and 118-119	0.39	1.32	3.41
15	Other / random coil	48.39	51.01	1.05

Supplementary Table S3 – Definitions of 106-126 superclusters:

Cluster	Cluster Description
1	Predominately helical
2	N-terminal and central domains helical, C-terminal domain not helical
3	C-terminal and central domains helical, N-terminal domain not helical
4	β-hairpin with a turn at res 114-115
5	β-hairpin with a turn at res 113-114
6	β-hairpin with a turn at res 116-117
7	β-hairpin with a turn at res 112-114
8	β-hairpin with a turn at res 115-116
9	Double β-hairpin with turns at res 113-114 and 118-120
10	Double β-hairpin with turns at res 114-115 and 118-120
11	Double β-hairpin with turns at res 112-114 and 118-120
12	β-hairpin with a turn at res 119-120
13	β-hairpin with a turn at res 118-119
14	β-hairpin with a turn at res 118-120
15	Triple β-hairpin with turns at res 113-114, res 118-120 and 122-124
16	Triple β-hairpin with turns at res 114-115, res 118-120 and 122-124
17	Triple β-hairpin with turns at res 116-117, res 118-120 and 122-124
18	Triple β-hairpin with turns at res 112-114, res 118-120 and 122-124
19	Triple β-hairpin with turns at res 115-116, res 118-120 and 122-124
20	Double β-hairpin with turns at res 114-115 and res 122-124 (not turn at res 118-120)
21	Double β-hairpin with turns at res 113-114 and res 122-124 (not turn at res 118-120)
22	Double β-hairpin with turns at res 116-117 and res 122-124 (not turn at res 118-120)
23	Double β-hairpin with turns at res 112-114 and res 122-124 (not turn at res 118-120)
24	Double β-hairpin with turns at res 115-116 and res 122-124 (not turn at res 118-120)
25	Double β-hairpin with turns at res 118-120 and res 122-124 (not turn at any places between res 7-12)
26	Central region helical but N- and C-terminal regions not helical
27	N-terminal region helical but central and C-terminal region not helical
28	C-terminal region helical but central and N-terminal region not helical
29	Unstructured or other

Supplementary Table S4 – Populations of superclusters for the 106-126 and 106-126 $A_{117}V$ peptide simulations

Rule order	Cluster (as per Supplementary Table S3)	106-126 (%)	106-126 $A_{117}V$ (%)	Change resulting from $A_{117}V$ mutation
1	Predominately helical	24.83	21.45	0.86
2	N-terminal and central domains helical, C-terminal domain not helical	12.89	9.63	0.75
3	C-terminal and central domains helical, N-terminal domain not helical	0.17	0.35	2.04
27	N-terminal region helical but central and C-terminal region not helical	5.64	7.01	1.24
28	C-terminal region helical but central and N-terminal region not helical	1.25	1.73	1.39
26	Central region helical but N- and C-terminal regions not helical	5.69	4.29	0.75
7	β-hairpin with a turn at res 112-114	0.17	0.13	0.80
5	β-hairpin with a turn at res 113-114	0.33	1.11	3.34
4	β-hairpin with a turn at res 114-115	0.27	1.21	4.41
8	β-hairpin with a turn at res 115-116	0.08	0.21	2.58
6	β-hairpin with a turn at res 116-117	0.33	0.09	0.28
13	β-hairpin with a turn at res 118-119	1.28	1.06	0.83
14	β-hairpin with a turn at res 118-120	0.06	0.05	0.89
12	β-hairpin with a turn at res 119-120	0.21	0.80	3.87
11	Double β-hairpin with turns at res 112-114 and 118-120	0.03	0.01	0.25
9	Double β-hairpin with turns at res 113-114 and 118-120	0.19	1.13	5.83
10	Double β-hairpin with turns at res 114-115 and 118-120	0.01	0.19	14
23	Double β-hairpin with turns at res 112-114 and res 122-124 (not turn at res 118-120)	0.02	0.03	1.33
21	Double β-hairpin with turns at res 113-114 and res 122-124 (not turn at res 118-120)	0.04	0.02	0.50
20	Double β-hairpin with turns at res 114-115 and res 122-124 (not turn at res 118-120)	0.07	0.5	8.09
24	Double β-hairpin with turns at res 115-116 and res 122-124 (not turn at res 118-120)	0.03	0.01	0.25
22	Double β-hairpin with turns at res 116-117 and res 122-124 (not turn at res 118-120)	0.05	0.14	3.00
25	Double β-hairpin with turns at res 118-119 and res 122-124 (not turn at any places between res 112-117)	0.33	0.33	1.00
18	Triple β-hairpin with turns at res 112-114, res 118-120 and 122-124	0.00	0.04	n/a
15	Triple β-hairpin with turns at res 113-114, res 118-120 and 122-124	0.0	0.13	n/a
16	Triple β-hairpin with turns at res 114-115, res 118-120 and 122-124	0.02	0.06	3.00
19	Triple β-hairpin with turns at res 115-116, res 118-120 and 122-124	0.00	0.01	n/a
17	Triple β-hairpin with turns at res 116-117, res 118-120 and 122-124	0.00	0.00	n/a
29	Unstructured or other	46.01	48.19	1.05

Supplementary Figure S1

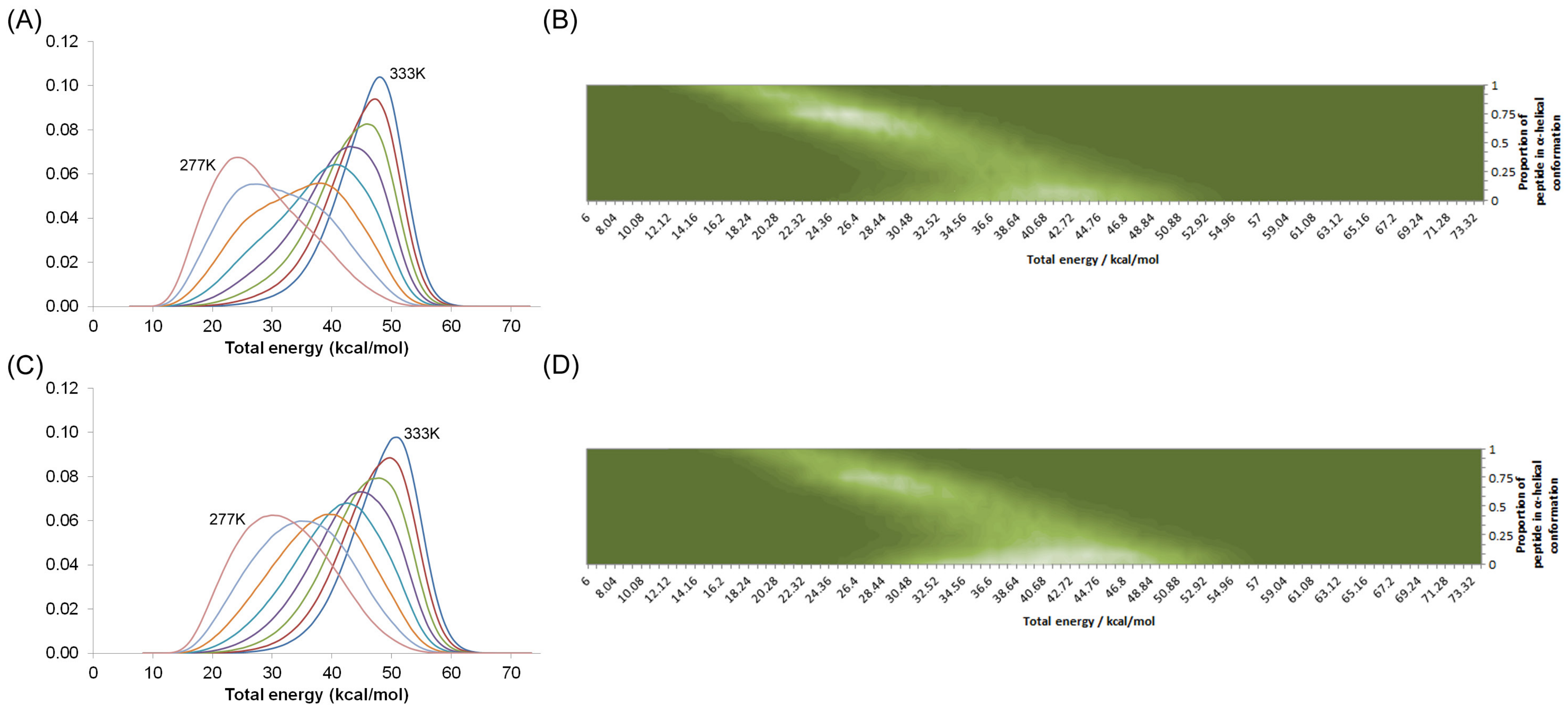

Supplementary Figure S1: (A) Energy histograms, as a function of temperature, produced from the first replicate simulation of the 109-122 peptide. Lower temperatures are associated with lower energies. (B) 2D histogram of energy verses α-helical content for 109-122 peptides at temperatures of 284 K or 292 K across all three replicate simulations. The majority of peptides segregate into two groups associated with high helical content or with essentially zero helical content. (C) Energy histograms, as a function of temperature, produced from the first replicate simulation of the 109-122 $A_{117}V$ peptide. Lower temperatures are associated with lower energies but the energy of the lower temperature states is closer to those of the higher temperature states. (D) 2D histogram of energy verses α-helical content for 109-122 $A_{117}V$ peptides at temperatures of 284 K or 292 K across all three replicate simulations. The majority of peptides segregate into two groups associated with high helical content or with essentially zero helical content, but the group at zero helical content is increased in abundance compared to the wildtype peptide. It also extends to lower energies and the peptides populating such states are predominately in β-hairpin conformations.

Supplementary Figure S2

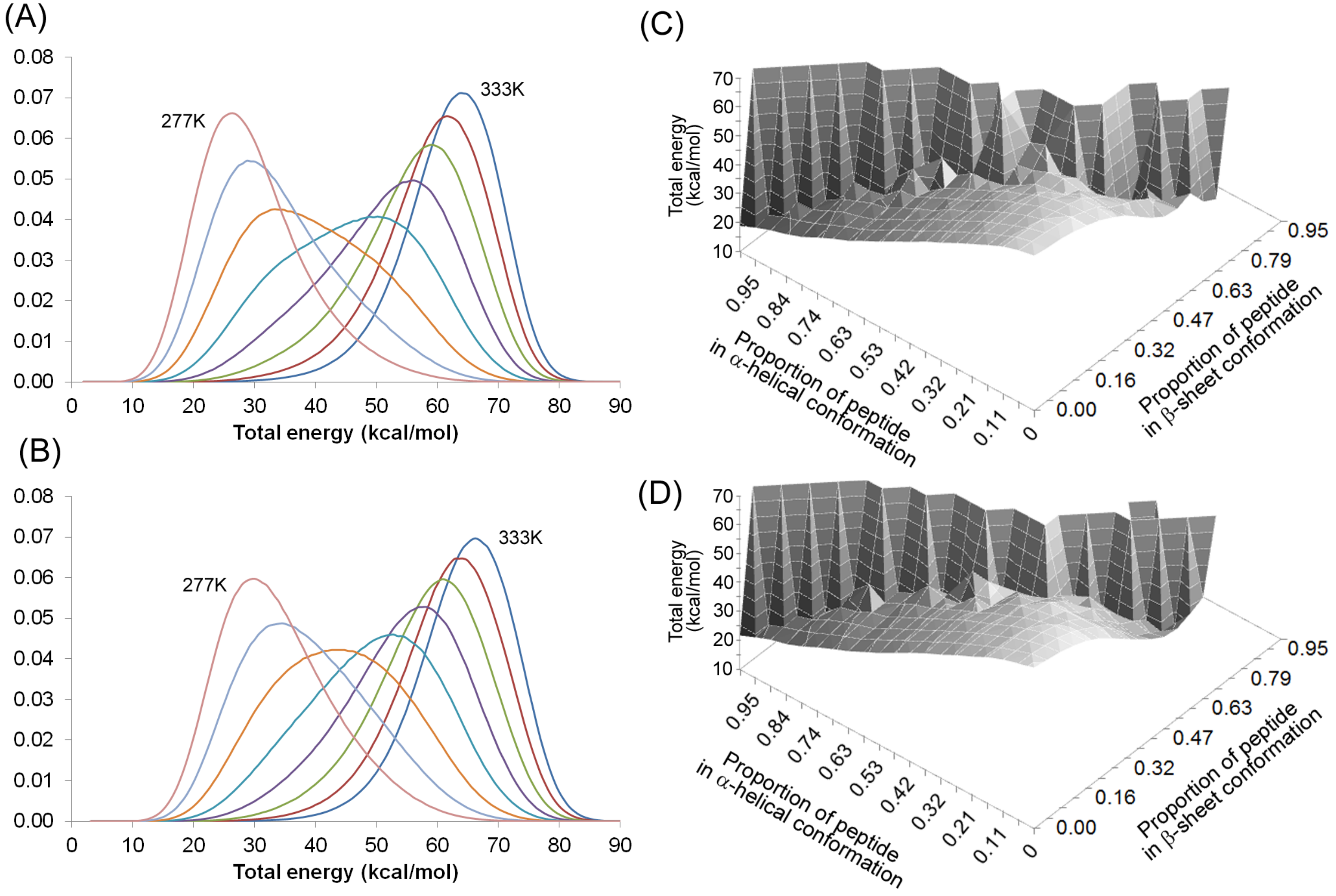

Supplementary Figure S2 (A) Energy histograms, as a function of temperature, produced from the first replicate simulation of the 106-126 peptide. Lower temperatures are associated with lower energies. (B) Energy histograms, as a function of temperature, produced from the first replicate simulation of the 106-126 $A_{117}V$ peptide. Lower temperatures are associated with lower energies but the energy of the lower temperature states is increased relative to those at higher temperatures. (C) Total energy profile of the 106-126 peptide as a function of the fraction of peptide in helical conformation and in β-strand conformation. Energy minima are located at high levels of helical content as well as high levels of β-strand content, corresponding to β-hairpin structures. (D) Total energy profile of the 106-126 $A_{117}V$ peptide as a function of the fraction of peptide in helical conformation and in β-strand conformation. Energy minima are located at high levels of helical content as well as high levels of β-sheet content, corresponding to β-hairpin structures.

Supplementary Figure S3

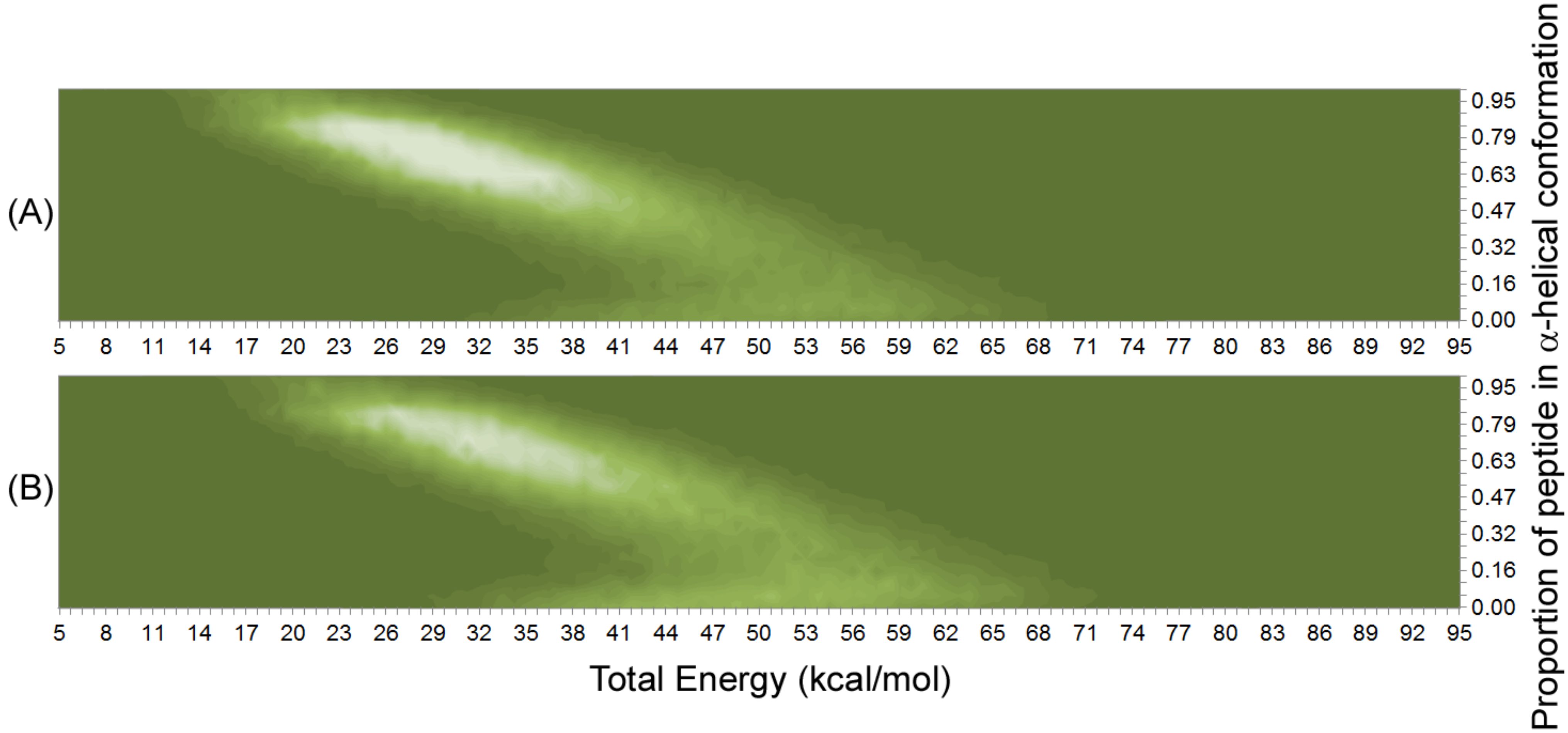

Supplementary Figure S3: (A) and (B) 2D histogram of energy verses α-helical content for the wildtype 106-126 peptide and the $A_{117}V$ mutant peptide respectively, including structures generated at simulation temperatures of 284 K and 292 K across all three replicate simulations. The majority of peptides are associated with high helical content, but the $A_{117}V$ mutation decreases the abundance of structures having high helical content.

Supplementary table S5: Summary of all multi-chain simulations performed during this work.

<table>
<tr><th>Peptide</th><th>Temp / K</th><th>Run no</th><th>Structural content (a)</th><th>Oligomerisation graph (b)</th><th>Final state (c)</th></tr>
<tr><td>109-122</td><td>293</td><td>1</td><td>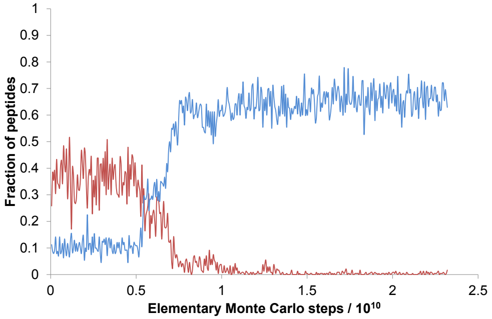
</td><td>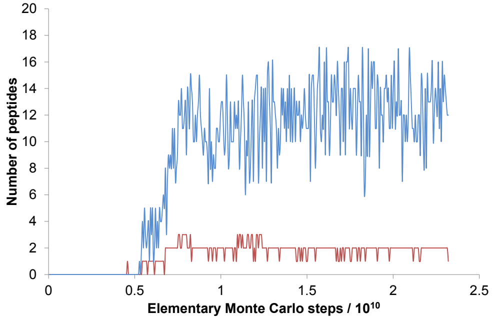
</td><td>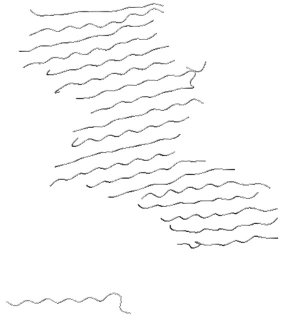</td></tr>
<tr><td>109-122</td><td>293</td><td>2</td><td>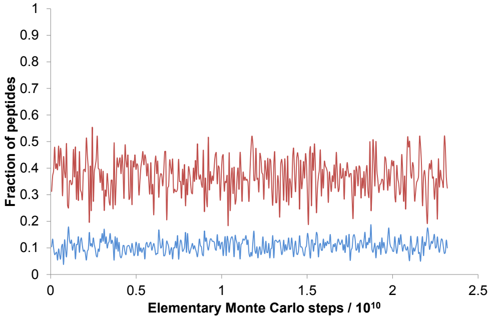
</td><td>No detectable oligomerisation / fibrillisation</td><td>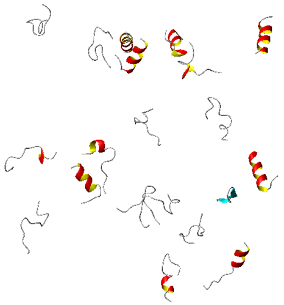</td></tr>
<tr><td>109-122</td><td>293</td><td>3</td><td>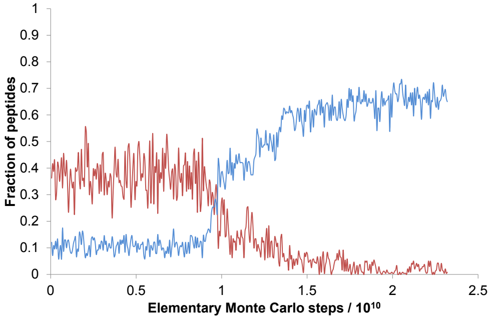
</td><td>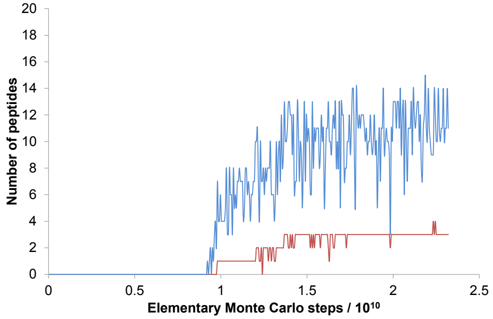
</td><td>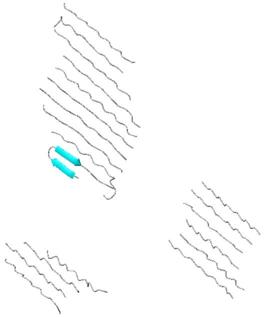</td></tr>
</table>

Peptide	Temp / K	Run no	Structural content [(a)]	Oligomerisation graph [(b)]	Final state [(c)]
109-122	303	1	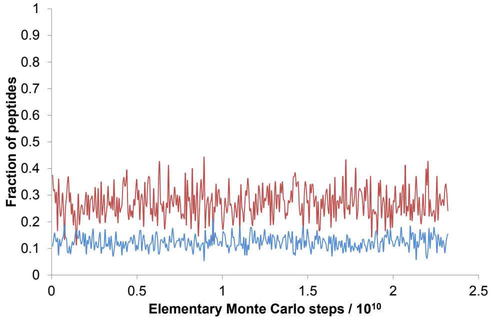	No detectable oligomerisation / fibrillisation	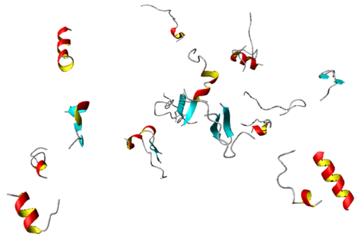
109-122	303	2	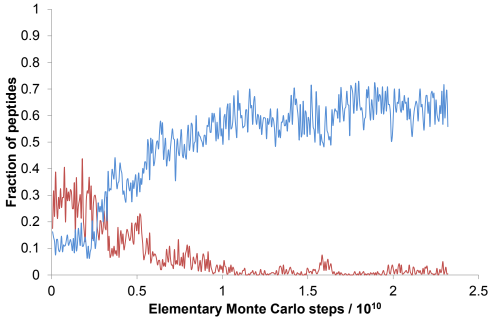	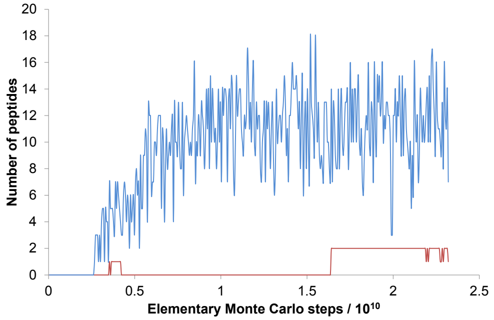	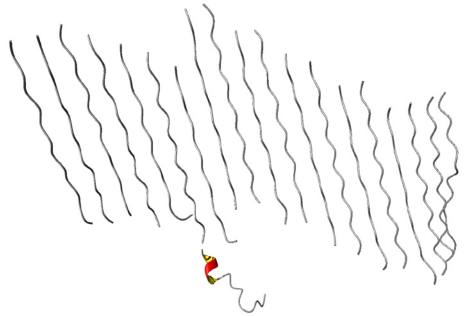
109-122	303	3	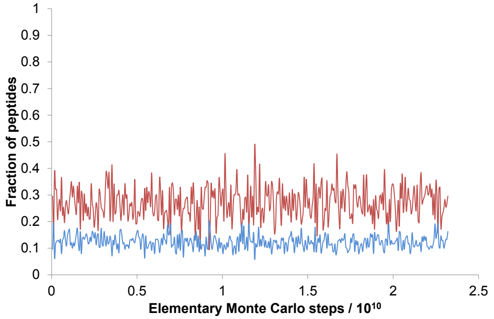	No detectable oligomerisation / fibrillisation	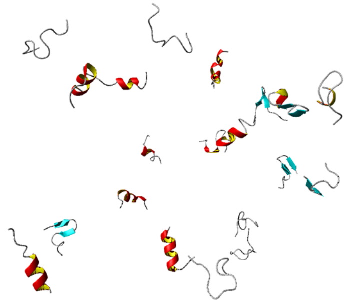

Peptide	Temp / K	Run no	Structural content [(a)]	Oligomerisation graph [(b)]	Final state [(c)]
109-122 $A_{117}V$	293	1	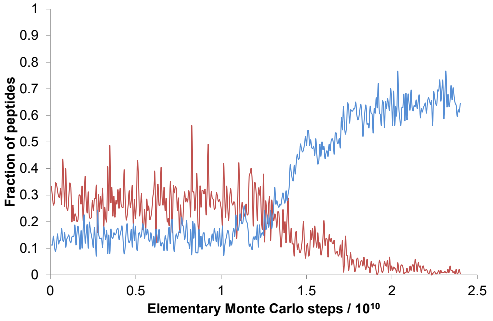	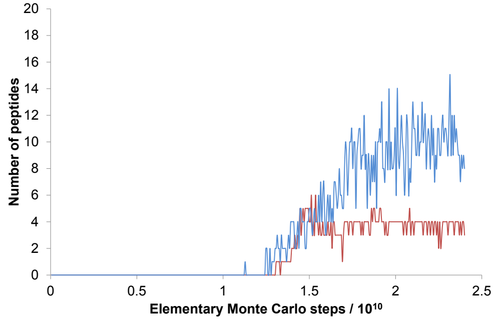	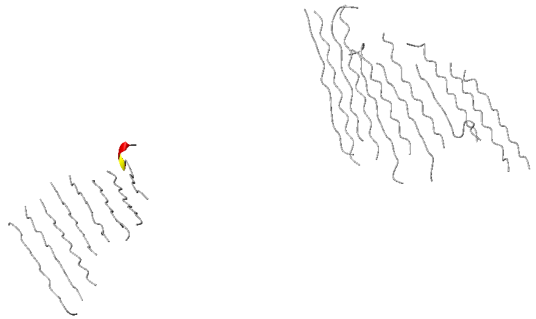
109-122 $A_{117}V$	293	2	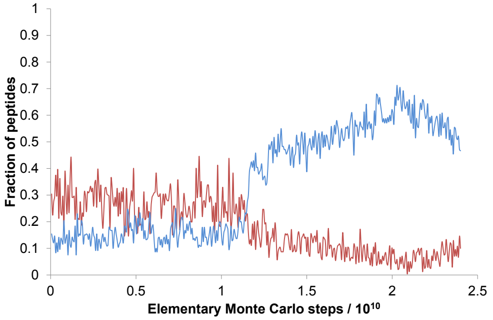	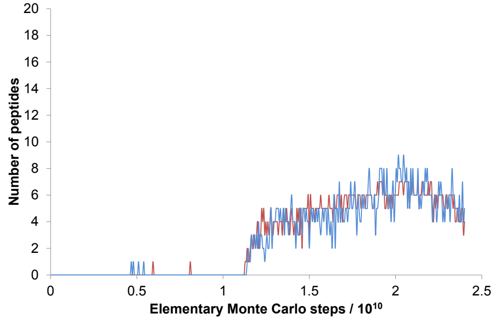	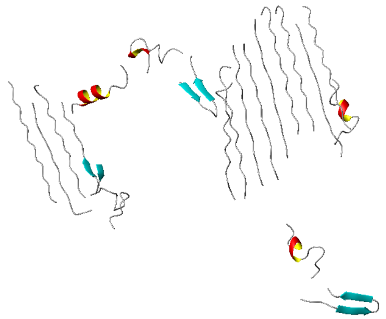
109-122 $A_{117}V$	293	3	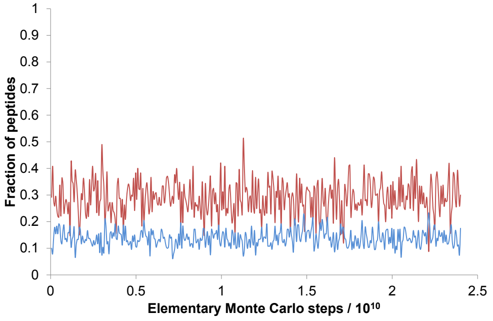	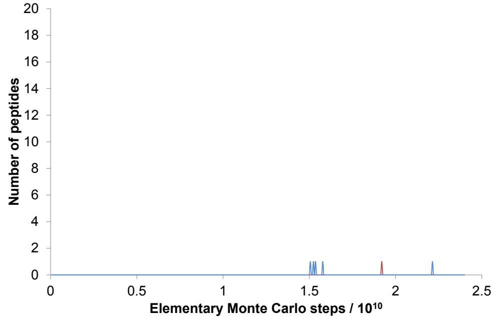	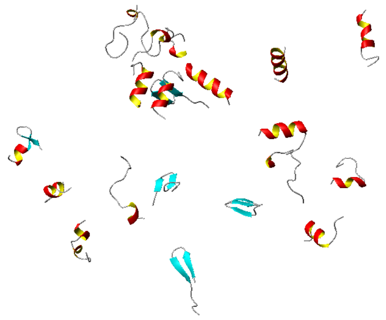

Peptide	Temp / K	Run no	Structural content [(a)]	Oligomerisation graph [(b)]	Final state [(c)]
109-122 $A_{117}V$	303	1	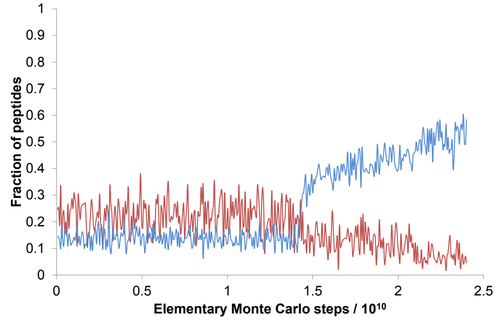	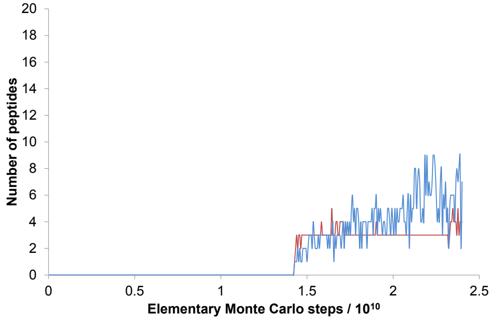	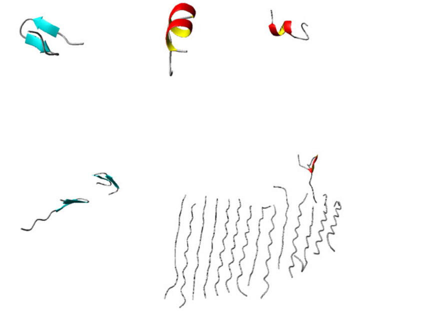
109-122 $A_{117}V$	293	2	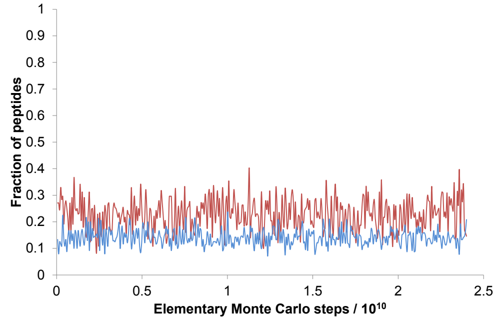	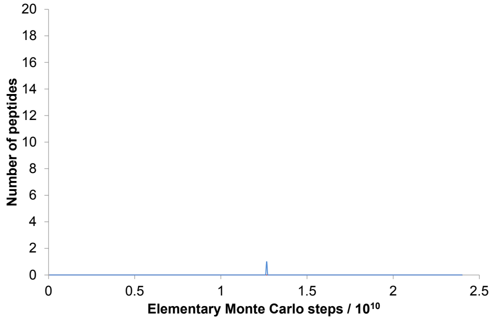	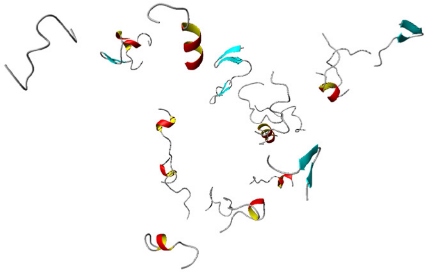
109-122 $A_{117}V$	293	3	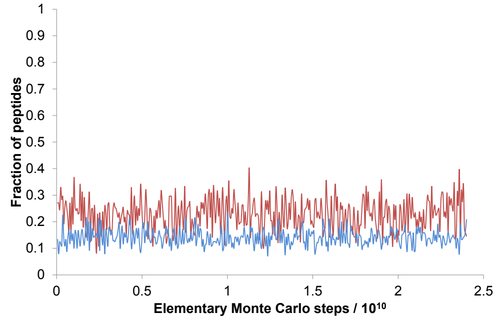	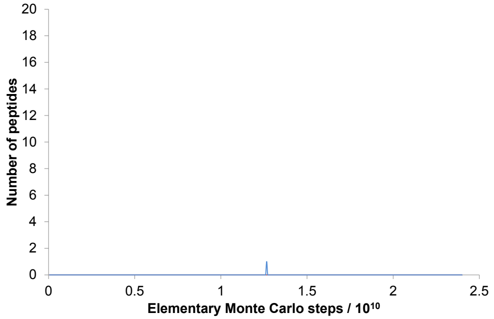	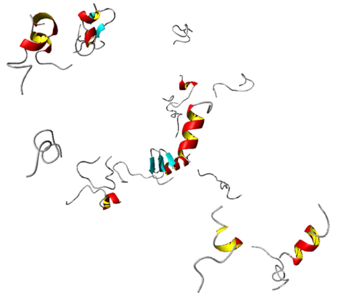

<table>
<tr><th>Peptide</th><th>Temp / K</th><th>Run no</th><th>Structural content (a)</th><th>Oligomerisation graph (b)</th><th>Final state (c)</th></tr>
<tr><td>106-126</td><td>293</td><td>1</td><td>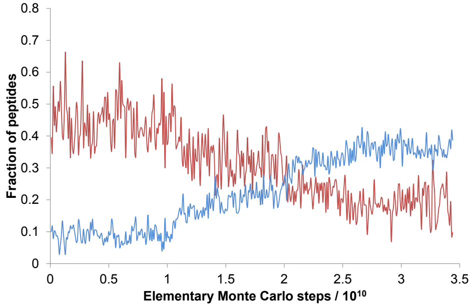</td><td>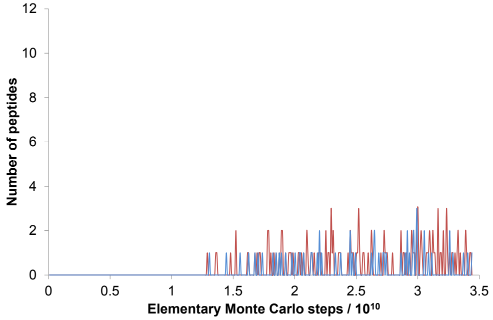</td><td>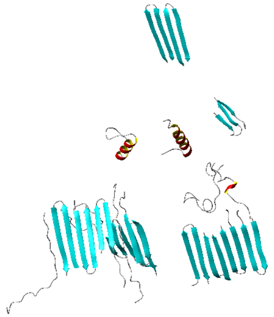</td></tr>
<tr><td>106-126</td><td>293</td><td>2</td><td>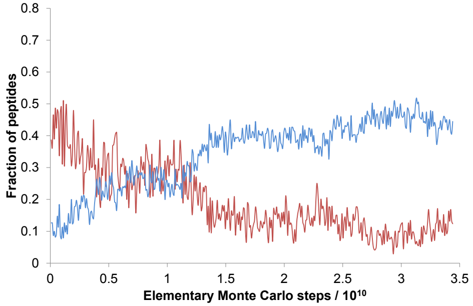</td><td>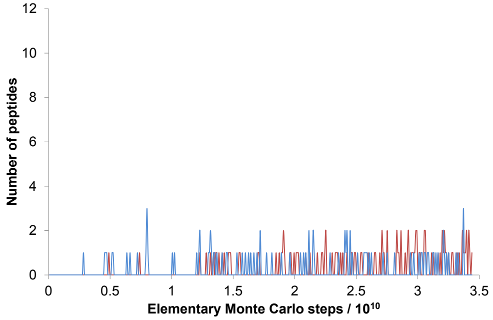</td><td>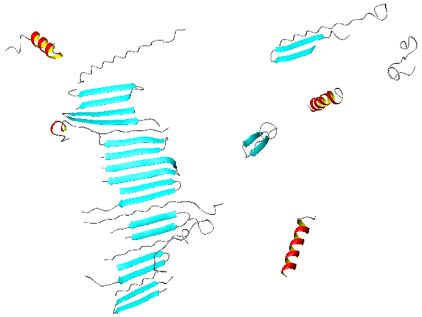</td></tr>
<tr><td>106-126</td><td>303</td><td>1</td><td>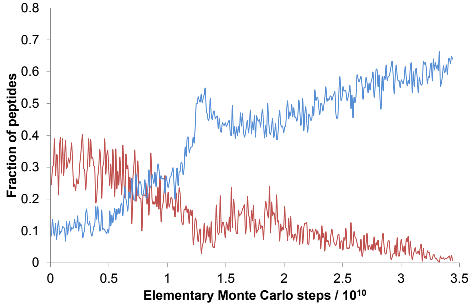</td><td>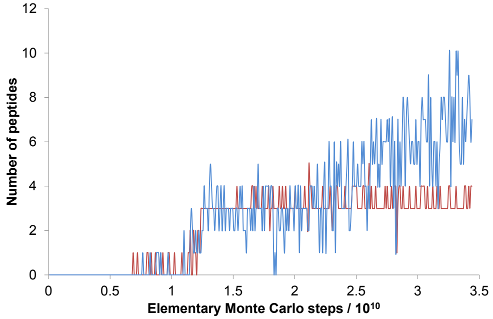</td><td>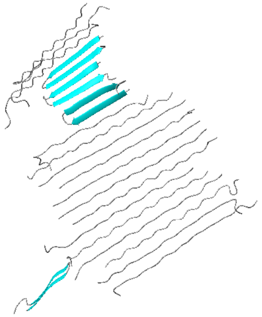</td></tr>
</table>

Peptide	Temp / K	Run no	Structural content [(a)]	Oligomerisation graph [(b)]	Final state [(c)]
106-126	303	2	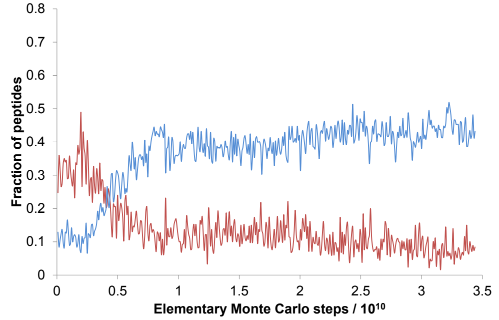	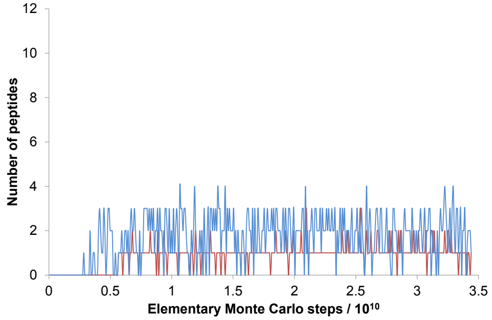	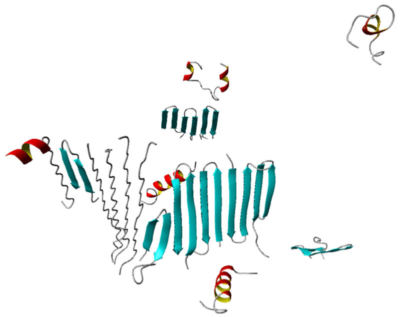
106-126 $A_{117}V$	293	1	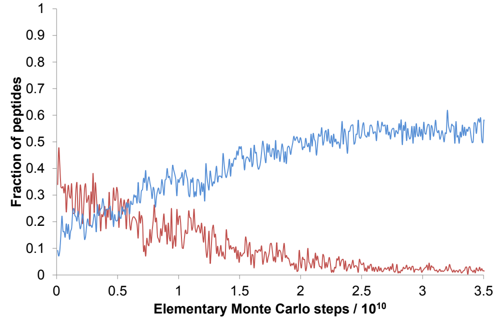	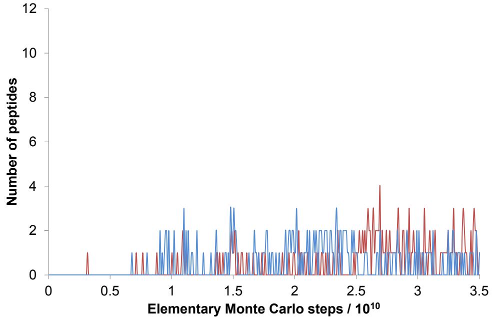	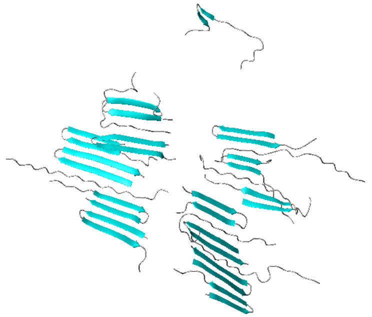
106-126 $A_{117}V$	293	2	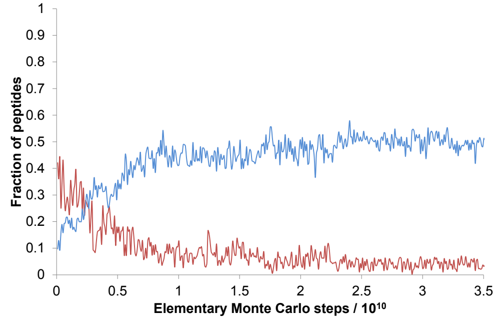	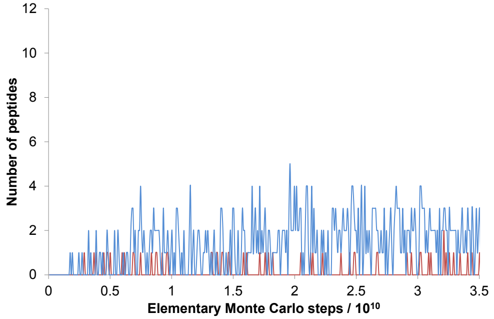	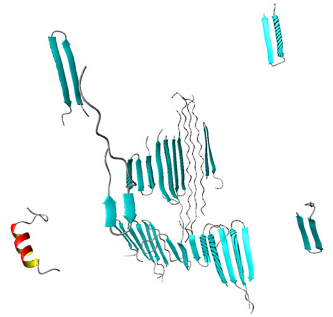

Peptide	Temp / K	Run no	Structural content (a)	Oligomerisation graph (b)	Final state (c)
106-126 $A_{117}V$	303	1	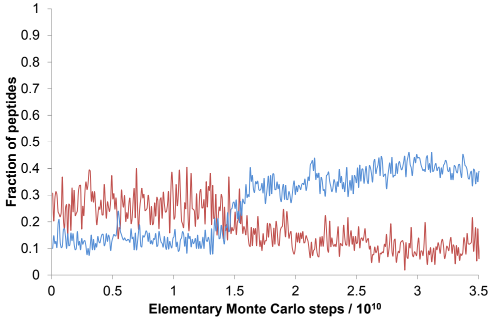	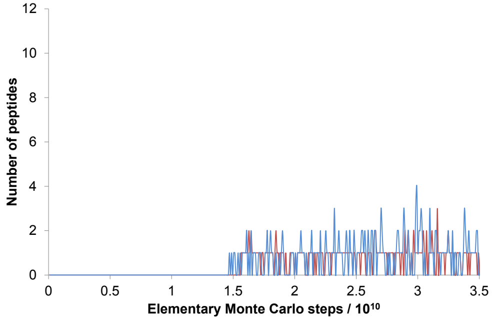	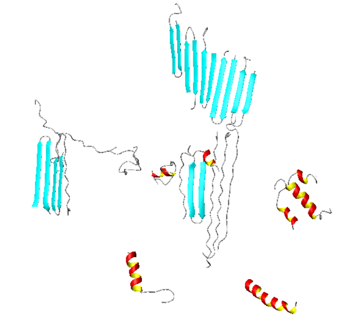
106-126 $A_{117}V$	303	2	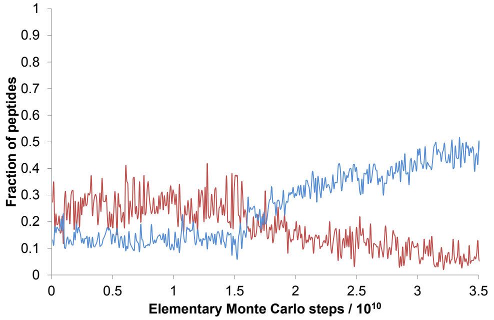	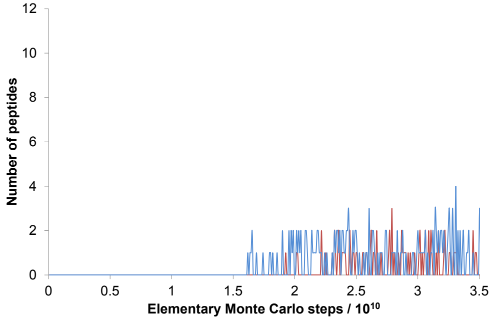	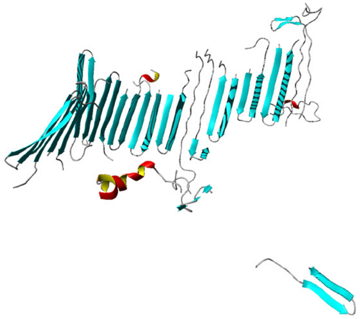

(a) In all graphs of structural content, the red traces indicate the amount of α-helical structure in the total population of peptides, whilst the blue traces relate to the amount of β-sheet structure in the population. (b) In all graphs of the number of peptide chains contained in oligomeric/fibrillar assemblies, the red lines relate to chains in parallel β-strand conformation whilst the blue lines relate to chains in anti-parallel β-strands. (c) The final state of the population represents the end of the simulation period only and is included for illustrative purposes only; it is not intended to imply that the states shown represent equilibrium/steady state conditions since further simulations would inevitably alter the distribution of monomer-to-multimeric assemblies, and potentially the structure of fibrillar forms, in any given simulation.

Supplementary Figure S4

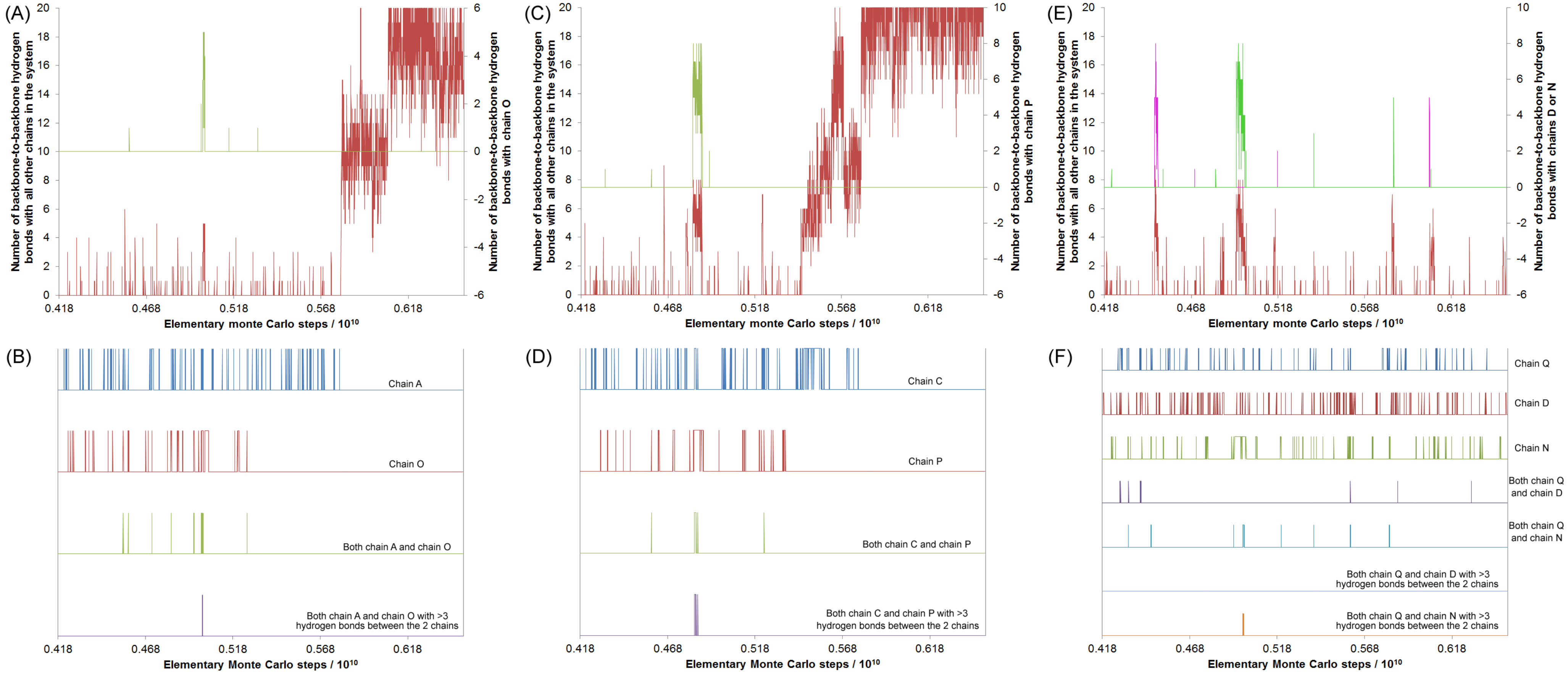

Detailed plots of the pre- and mid-association phase from simulation of 20 copies of the 109-122 peptide at 293 K (replicate 1). Before about 0.54 x 10^{10} steps the system is not fibrillizing and no nucleus has formed. After this point many chains become involved in a growing fibril driven by extensive hydrogen bond formation (A) The number of hydrogen bonds formed between backbone atoms of chain A and backbone atoms of any other of the chains in the system (red) or specifically with backbone atoms of chain O (green). (B) Simulation frames in which β-hairpin structures are formed by chain A, chain O, where both chains form β-hairpin structures concomitantly or where there are >3 hydrogen bonds between two chains both of which are in β-hairpin conformation. (C) The number of hydrogen bonds formed between backbone atoms of chain C and backbone atoms of any other of the chains in the system (red) or specifically with backbone atoms of chain P (green). (D) Simulation frames in which β-hairpin structures are formed by chain C, chain P, where both chains form β-hairpin structures concomitantly or where there are >3 hydrogen bonds between two chains both of which are in β-hairpin conformation. (E) The number of hydrogen bonds formed between backbone atoms of chain Q and backbone atoms of any other of the chains in the system (red) or specifically with backbone atoms of chain D (blue) or chain N (green). (F) Simulation frames in which β-hairpin structures are formed by chain Q, chain D, chain N, where chains form β-hairpin structures concomitantly or where there are >3 hydrogen bonds between two chains both of which are in β-hairpin conformation.
